# PABPN1 Couples the Polyadenylation and Translation of Maternal Transcripts to Mouse Oocyte Meiotic Maturation

**DOI:** 10.1002/advs.202500048

**Published:** 2025-04-23

**Authors:** Xing‐Xing Dai, Yu‐Ke Wu, Shuai‐Bo Pi, Feng‐Jie Hu, Yun‐Wen Wu, Hang Qi, Zhiyi Li, Zhi‐Yan Jiang, Long‐Wen Zhao, Heng‐Yu Fan

**Affiliations:** ^1^ Department of Obstetrics and Gynecology Center for Reproductive Medicine the Fourth Affiliated Hospital of School of Medicine and International School of Medicine International Institutes of Medicine Zhejiang University Yiwu 322000 China; ^2^ Life Sciences Institute Zhejiang University Hangzhou 310058 China; ^3^ Department of Computer Science and Engineering College of Engineering The Ohio State University‐Columbus; ^4^ Zhejiang Key Laboratory of Precise Protection and Promotion of Fertility Sir Run Run Shaw Hospital School of Medicine Zhejiang University Hangzhou 310016 China; ^5^ Center for Biomedical Research Shaoxing Institute Zhejiang University Shaoxing 312000 China

**Keywords:** female fertility, meiosis, oocyte maturation, RNA stability, RNA‐binding protein, translation activity

## Abstract

During oocyte meiosis, maternal transcript polyadenylation is crucial for regulating mRNA stability and translation, which are essential for oocyte maturation. Polyadenylate‐binding protein nuclear 1 (PABPN1) plays a key role in regulating mRNA splicing and polyadenylation in somatic cells and growing oocytes. However, its potential function in regulating the meiotic maturation of fully grown oocytes remains unknown. This study reports that selective *Pabpn1* knockout in growing mouse oocytes using *Zp3‐Cre* do not affect folliculogenesis but prevented germinal vesicle breakdown in fully grown oocytes, impaired CDK1 activation, and resulted in abnormal spindle formation and chromosome misalignment. The results of poly(A)‐inclusive full‐length RNA isoform sequencing (PAIso‐seq) and transcriptome sequencing revealed that PABPN1 coordinates meiotic maturation‐coupled polyadenylation and degradation of maternal mRNAs, which are key factors of maturation‐promoting factor (MPF) and deadenylation mediators, such as B‐cell translocation gene‐4 (BTG4), ensuring proper meiotic progression. The results of rescue experiments indicate these functions of PABPN1 are mediated by its key domains, which interact with poly(A) polymerase and recruit target mRNAs. This study highlighted the physiological importance of cytoplasmic PABPN1 in mammalian oocyte maturation by integrating maternal transcript polyadenylation, translation, and degradation.

## Graphical abstract

This study reveals that cytoplasmic PABPN1 is essential for mouse oocyte meiotic maturation by coordinating polyadenylation, translation, and degradation of maternal mRNAs. *Pabpn1* knockout disrupts CDK1 activation, spindle formation, and chromosome alignment by impairing maturation‐promoting factor (MPF) regulation and BTG4‐mediated deadenylation. PAIso‐seq and rescue experiments show that PABPN1's RNA‐binding domains coordinate poly(A) polymerase and target mRNAs to ensure meiotic progression.

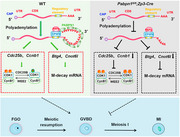

## 1 Introduction

Maternal transcript polyadenylation is a crucial regulatory mechanism during oocyte meiosis, particularly during the transition from meiotic arrest to maturation.^[^ ^1^ ^]^ This process is essential for oocyte maturation and early embryonic development because it regulates protein synthesis. The length of the poly(A) tail directly affects mRNA stability, extends transcript lifespan, and prevents rapid degradation, ensuring sustained expression of essential proteins.^[^ ^2^ ^]^

In recent years, polyadenylation tail sequencing technologies such as PAIso‐seq and 3′ end sequencing have made progress.^[^ ^3^, ^4^, ^5^ ^]^ Research conducted using PAIso‐seq technology has revealed that during the human oocyte‐embryo transition, extensive remodeling of maternal mRNA poly(A) tails occurs, including variations in length and incorporation of non‐A residues, which are essential for the first cleavage of the human embryo.^[^ ^6^, ^7^ ^]^ However, over 70% of maternal transcripts contain only poly(A) tails with no non‐A residue insertions during oocyte meiosis.^[^ ^6^ ^]^ Research on key regulatory factors and networks that promote cytoplasmic polyadenylation during oocyte meiosis remains limited.

PABPN1 is a crucial poly(A) tail‐binding protein involved in the addition and stabilization of poly(A) tails. By binding to the poly(A) tail, it protects transcripts from degradation and influences their translation efficiency.^[^ ^8^, ^9^ ^]^ Mutations in PABPN1 are associated with neurodegenerative diseases and oculopharyngeal muscular dystrophy, as PABPN1 mutations may lead to abnormal poly(A) tails that affect transcript stability and translation.^[^ ^10^, ^11^ ^]^ PABPN1 is a crucial regulatory factor for the clearance of maternal mRNA. It depends on zygotic genome activation during early embryonic development in mice and plays an essential role in embryogenesis.^[^ ^12^ ^]^ In growing mouse oocytes, PABPN1 promotes the formation of nuclear polyadenylation domains (NPAD) through liquid–liquid phase separation, where it interacts with the splicing factors serine/arginine‐rich splicing factor 2 (SRSF2) and CPSF4 to regulate mRNA processing and stability.^[^ ^13^ ^]^ This process is crucial for the development of mouse oocytes, and the loss of PABPN1 results in female infertility. As oocyte meiosis resumes and germinal vesicles break down, PABPN1 is dispersed into the cytoplasm. However, their physiological functions and regulatory mechanisms in the cytoplasm remain unclear.

Members of the poly(A) polymerase complex, such as CPSF4 and PAPα, play crucial roles in cells, collectively regulating the addition and stability of the poly(A) tail.^[^ ^14^, ^15^ ^]^ Research indicates that CPSF4 is essential for cytoplasmic polyadenylation of mRNA and normal oocyte maturation.^[^ ^16^ ^]^ PAPα is a critical enzyme in mediating cytoplasmic mRNA polyadenylation in mouse oocytes. Inhibition of PAPα activity impairs cytoplasmic polyadenylation and translation of maternal transcripts, hindering meiotic cell‐cycle progression.^[^ ^17^ ^]^ Although the roles of these regulatory factors have been reported, the mechanisms by which they collectively promote cytoplasmic polyadenylation require further investigation.

Oocyte degradation of maternal mRNA is another critical physiological event.^[^ ^18^ ^]^ Maternal transcript degradation is tightly regulated at specific stages, particularly at the end of meiosis and during early embryonic development.^[^ ^7^ ^]^ BTG4 is a key regulatory factor in the maternal‐to‐zygotic transition of mouse oocytes and triggers maternal mRNA degradation through its interaction with the CCR4‐NOT deadenylase complex and eukaryotic translation initiation factor 4E (eIF4E), which is crucial for female fertility.^[^ ^19^, ^20^ ^]^ The catalytic subunit CNOT6L of the CCR4‐NOT complex interacts with zinc finger protein 36 like 2 (ZFP36L2) in mouse oocytes to mediate maternal mRNA degradation, coupling this process with the meiotic cell cycle. The absence of these genes can lead to meiotic defects and infertility.^[^ ^21^ ^]^ How these factors, in coordination with polyadenylation‐related proteins, regulate the meiotic process of oocytes and maintain the balance between mRNA stability and translation, remains a key unresolved scientific question in the study of oocyte meiosis.

In this study, we reveal that PABPN1 facilitates cytoplasmic polyadenylation and translation of maternal mRNAs to promote the resumption of meiotic processes in fully grown mammalian oocytes. In addition, PABPN1 regulates the molecular mechanism that couples maternal transcript polyadenylation, maternal mRNA degradation, and the meiotic process by modulating the polyadenylation of CCNB1 and deadenylation factor BTG4.

## 2 Results

### 2.1 PABPN1 is Expressed in Mouse Oocytes During Meiotic Maturation and is Essential for Female Fertility

**Figure 1:**
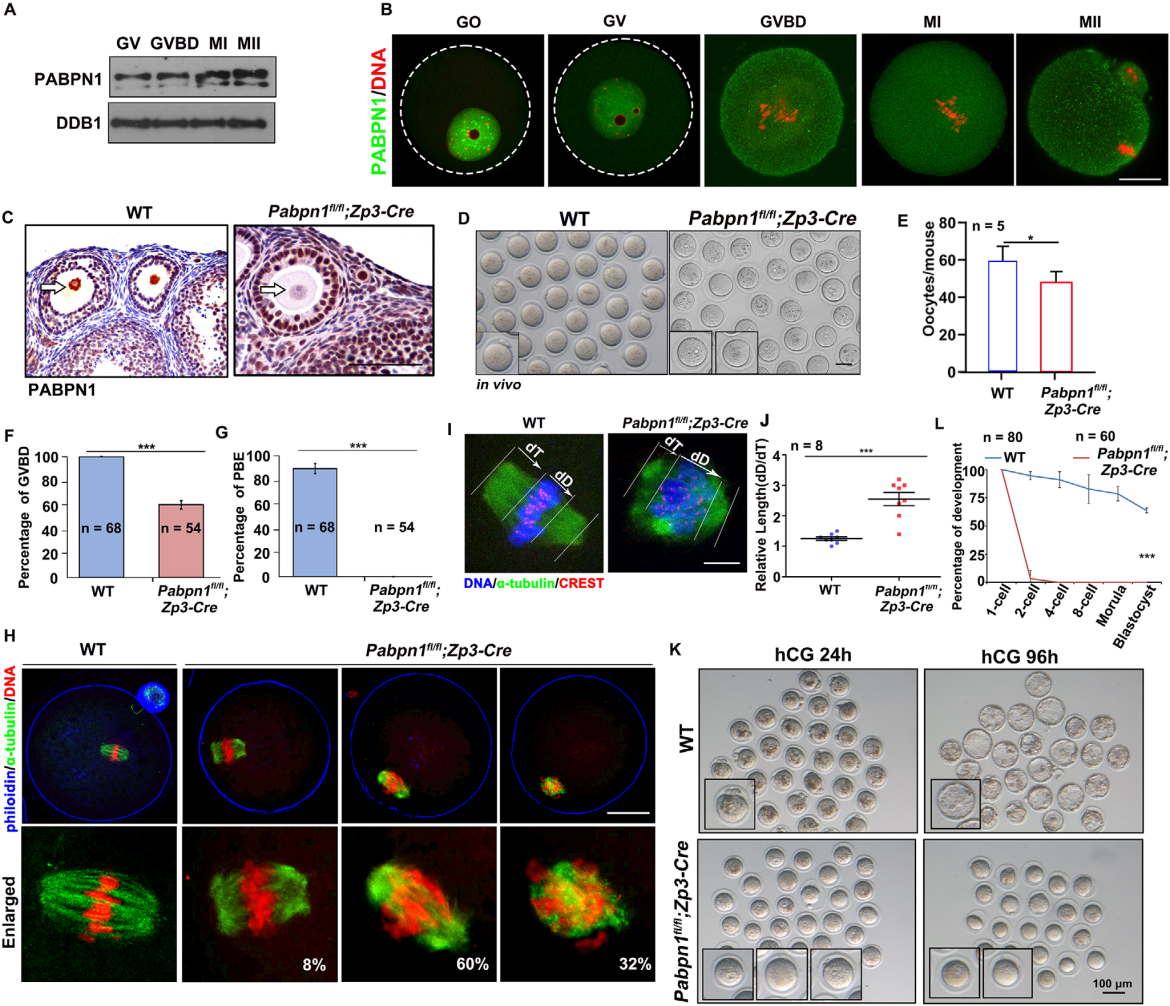
PABPN1 expression patterns and phenotypes in *Pabpn1^fl/fl^;Zp3‐Cre* mice. A) Western blotting results showing the expression of PABPN1 during meiotic maturation of oocytes. DDB1, with 200 oocytes per period, was used as a loading control. B) Immunofluorescence showing the localization and expression of PABPN1 in mouse oocytes from the growth stage (GO) to meiotic maturation stage (GVBD, MI, and MII). Scale bar: 20 µm. GO, growing oocyte; GV, germinal vesicle oocyte; GVBD, germinal vesicle breakdown; MI, metaphase I; MII, metaphase II. C) Immunohistochemistry results show that PABPN1 was effectively knocked out in oocytes from *Pabpn1^fl/fl^;Zp3‐Cre* mice at the primary‐follicle stage. PABPN1 expression in oocyte nuclei is indicated by the arrow. Scale bars represent 100 µm. D) Images of representative oocytes collected from WT and *Pabpn1^fl/fl^;Zp3‐Cre* mice at 16 h after hCG injection. Scale bar, 20 µm. E) Quantification of ovulated oocytes from wild‐type (WT) and *Pabpn1^fl/fl^;Zp3‐Cre* mice. Statistical significance was determined using two‐tailed Student's *t*‐tests, with indicating ^*^ *p* < 0.05. *n* = 5 females for each genotype. F,G) GVBD and polar body‐1 emission (PBE) in (D). Error bars represent the standard error of the mean (SEM). Statistical significance was determined using two‐tailed Student's *t‐*tests, with indicating ^***^ *p* < 0.001. The number of oocytes analyzed is shown (n). H) Immunofluorescence results showing spindles in WT and *Pabpn1^fl/fl^;Zp3‐Cre* mouse oocytes. The percentage of representative phenotypes/total oocytes observed is indicated in the corner. Scale bar, 20 µm. I,J) Measurement of chromosomes relative to spindle. dD, maximum chromosome span. dT indicates the maximum length between the DNA and spindle tip. The scattergram (J) shows the dD:dT ratios for WT and *Pabpn1^fl/fl^;Zp3‐Cre* mouse oocytes. The number of oocytes analyzed is indicated (n). Error bars indicate SEM. ^***^p < 0.001 by two‐tailed Student's *t‐*tests. Scale bar, 1 µm. K) Representative images of oviducts or uteri of embryos collected from WT and *Pabpn1^fl/fl^*; *Zp3*‐*Cre* mice at the specified time points following hCG injection. Scale bar = 100 µm. L) Embryo development was quantified in WT and *Pabpn1^fl/fl^;Zp3‐Cre* mice after mating with adult WT males, with analysis conducted at the specified stages. The number of embryos analyzed is denoted as (n). Error bars represent the standard error of the mean (SEM). Statistical significance was determined using two‐tailed Student's *t*‐tests, with indicating ^***^ *P* < 0.001. The number of zygotes analyzed is shown (n).

Western blotting revealed that PABPN1 is expressed in mouse oocytes during meiotic maturation (**Figure** **1**A; Figure S1A,B, Supporting Information). Immunofluorescence results showed that PABPN1 was localized in the nuclei of oocytes before the full growth stage and diffused into the cytoplasm after the meiotic resumption, as characterized by GVBD (Figure 1B; Figure S1A, Supporting Information). Notably, PABPN1 staining is more pronounced in the cytoplasm of fully grown GV oocytes than in growing GV oocytes (Figure 1B), which could suggest a distinct functional role for PABPN1 in the cytoplasm, potentially linked to the regulation of maternal mRNA during oocyte maturation.

To further investigate whether PABPN1 was necessary for oocyte meiotic maturation, we generated *Pabpn1*‐floxed mice and crossed them with *Zp3‐Cre* transgenic mice, enabling *Pabpn1* knockout in growing oocytes starting from the primary‐follicle stage (Figure S1C, Supporting Information). Immunohistochemistry showed that compared to wild‐type (WT) mice, PABPN1 was not detected in the oocytes of *Pabpn1^fl/fl^;Zp3‐Cre* mice (Figure 1C). *Pabpn1^fl/fl^;Zp3‐Cre* mice were sterile in an 8‐month fertility test. (Figure S1D, Supporting Information). The 4‐week‐old *Pabpn1^fl/fl^;Zp3‐Cre* mice ovulated oocytes normally 16 h after the superovulation treatment (Figure S1E, Supporting Information) and with oocyte numbers comparable to those of WT mice at the same age (Figure 1D,E).

WT ovulated oocytes mostly emitted the first polar body (PB1), whereas 40% of the *Pabpn1*‐null ovulated oocytes were still arrested at the GV stage, and the rest at metaphase I (MI) or pre‐MI stages without emitting PB1 (Figure 1F,G). We collected oocytes at the GVBD stage and analyzed their defects using α‐tubulin staining during oocyte meiotic maturation. Immunofluorescence results showed that the *Pabpn1*‐null oocytes contained distorted spindles (Figure 1H). To quantitatively assess chromosomal alignment in oocytes, we measured the width of condensed chromosomes relative to spindle length (Figure 1I). The results revealed that the chromosomes were significantly wider in *Pabpn1*‐null oocytes than in WT oocytes (Figure 1I,J).

We superovulated 4‐week‐old *Pabpn1^fl/fl^;Zp3‐Cre* females, mated them with WT males, and assessed fertilization rates by confocal microscopy of pronuclei formation after DNA staining (Figure S1F,G, Supporting Information). Most WT zygotes successfully formed pronuclei, whereas zygotes derived from *Pabpn1* knockout oocytes hardly formed pronuclei (Figure S1F,G, Supporting Information). Over 70% of the control zygotes developed to the blastocyst stage, whereas most zygotes derived from *Pabpn1^fl/fl^;Zp3‐Cre* females underwent 1‐cell arrest (Figure 1K,L). Therefore, *Pabpn1‐*null oocytes exhibit profound defects in GVBD and spindle assembly, with the majority failing to undergo fertilization.

### 2.2 *Pabpn1* Knockout Affects the In Vitro Meiotic Maturation of Mouse Oocytes

Fully grown oocytes within cumulus–oocyte complexes (COCs) were harvested from the ovaries of WT and *Pabpn1^fl/fl^;Zp3‐Cre* mice (Figure S2A, Supporting Information). The ovaries of the WT and *Pabpn1^fl/fl^;Zp3‐Cre* mice contained comparable numbers of COCs (Figure S2A,B, Supporting Information). No significant difference was observed in the size of fully grown oocytes between WT and *Pabpn1*‐null mice (Figure S2C, Supporting Information). DAPI staining showed that over 90% of the GV‐stage oocytes from COCs of WT and *Pabpn1^fl/fl^;Zp3‐Cre* mice had a surrounding nucleolus (SN) configuration of chromatin (Figure S2D,E, Supporting Information). These results suggested that *Pabpn1* knockout by *Zp3‐Cre* did not affect the growth of oocytes and that *Pabpn1‐*null oocytes could develop into the GV stage in vivo.

**Figure 2:**
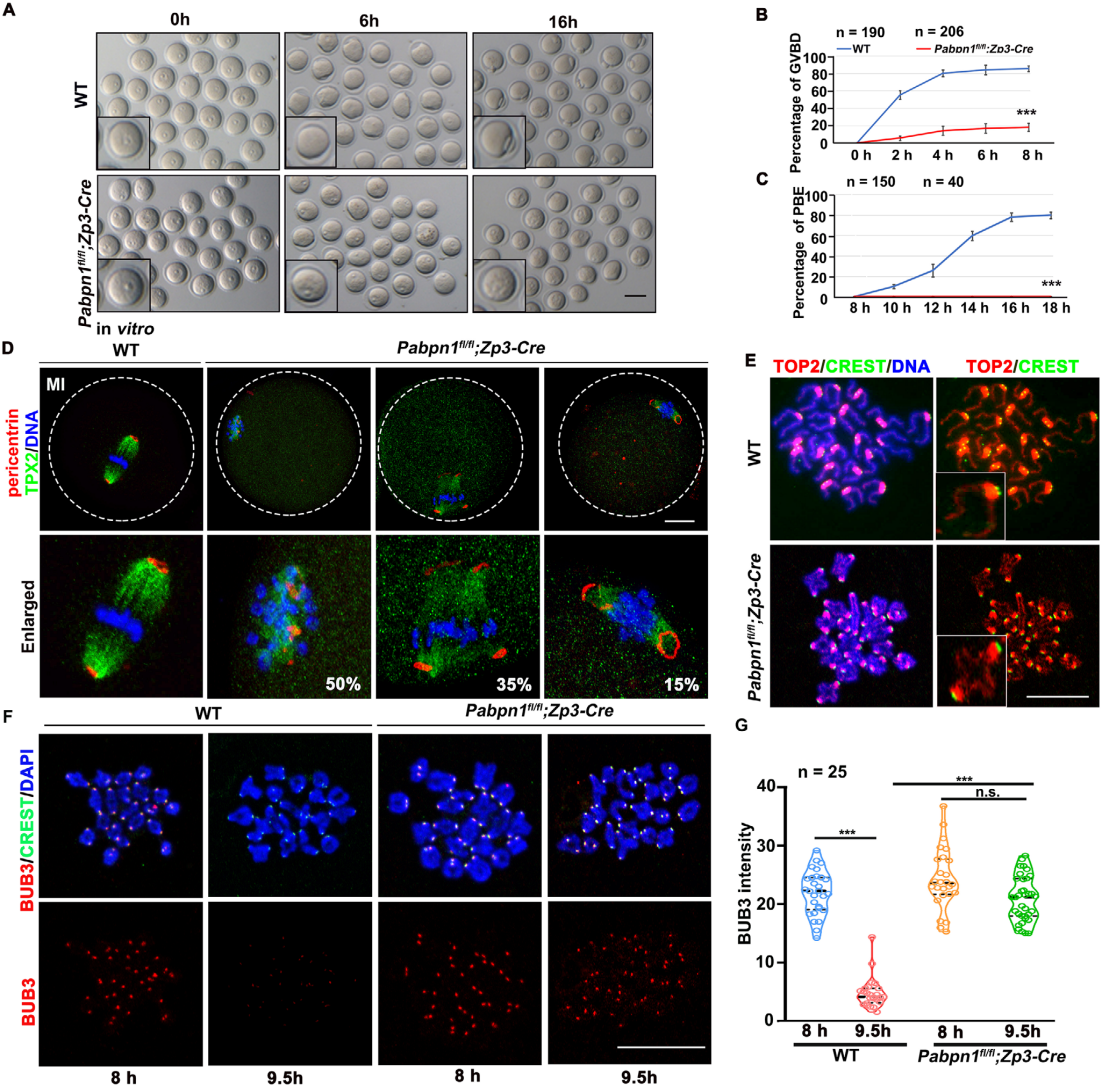
Effect of *Pabpn1* knockout on oocyte meiotic maturation. A) Representative images of WT and *Pabpn1^fl/fl^;Zp3‐Cre* mouse oocytes showing GVBD and PBE in oocytes cultured in vitro for 0, 6, and 16 h. Scale bar, 100 µm. B,C) Rate of GVBD (B) and PBE (C) in (A). Error bars indicate SEM. ^***^p < 0.001 (two‐tailed Student's *t‐*tests). The number of oocytes analyzed is shown (n). D) Immunofluorescence results show Pericentrin and TPX2 (microtubule nucleation factor) expression in cultured WT and *Pabpn1*‐null oocytes at the MI stage. Scale bar = 20 µm. E) Chromosomes spread from WT and *Pabpn1^fl/fl^;Zp3‐Cre* mice. The oocytes were cultured in vitro for 16 h. Immunofluorescence staining for topoisomerase II (TOP2) and CREST was performed to identify the chromosome arms and centromeres, respectively. Scale bar = 5 µm. F) Immunofluorescence of BUB3 on chromosome spreads prepared from WT and *Pabpn1*‐null oocytes at 8 and 9.5 h after culture. Scale bar, 5 µm. G) Quantification of the BUB3 signal intensity (F). The number of oocytes analyzed at each stage is indicated. Error bars indicate SEM. ^***^p < 0.001 by two‐tailed Student's *t‐*tests. n.s.: non‐significant.

We isolated GV oocytes from WT and *Pabpn1^fl/fl^;Zp3‐Cre* females and cultured them in vitro to examine the meiotic maturation process further. *Pabpn1*‐deficient oocytes exhibited severe GVBD defects and no PB1 emission (**Figure** **2**A–C). Although a small proportion of *the Pabpn1*‐null oocytes progressed to the GVBD stage (Figure 2A,B), the spindles of these oocytes were severely abnormal and contained distorted and multipolar spindles (Figure 2D). Pericentrin is a key component of the microtubule‐organizing center (MTOC), which is localized in the spindle's polar region in WT oocytes,^[^ ^21^ ^]^ whereas pericentrin clusters are not localized in the polar area of *Pabpn1*‐deficient oocytes (Figure 2D).

Chromosome spreading analysis revealed that after 16 h of in vitro culture, WT oocytes were in the diploid phase, whereas *Pabpn1*‐deficient oocytes remained in the tetraploid phase (Figure 2E). The anaphase‐promoting complex (APC) was inhibited by the spindle assembly checkpoint (SAC) at the prometaphase I to prevent meiotic errors.^[^ ^22^, ^23^ ^]^ When the cell cycle enters anaphase, the SAC marked by BUB3 is inactivated and disappears from the chromosome centromeres. In WT oocytes, BUB3 left the centromeres after the meiotic resumption, but remained on the centromeres of *Pabpn1*‐deficient oocytes (Figure 2F,G). In summary, a small proportion of *Pabpn1*‐deficient GVBD oocytes could not enter the anaphase of meiosis I.

### 2.3 GVBD Defects in *Pabpn1*‐Null Oocytes Could be Rescued with *Cdk1^T14A, Y15F^* mRNA Injection

**Figure 3:**
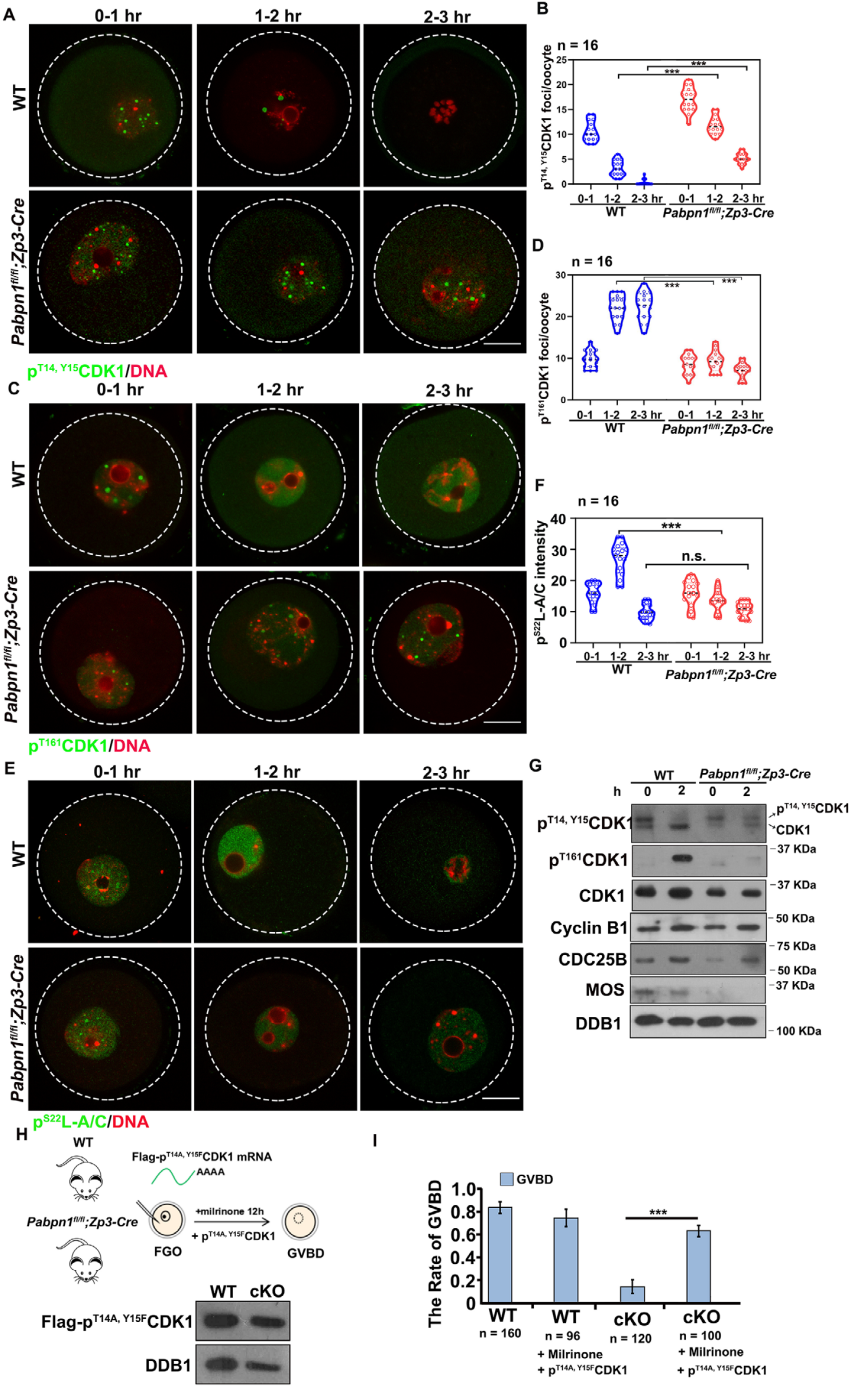
*Pabpn1*‐deleted oocytes exhibit meiotic abnormalities owing to the failure of CDK1 to undergo dephosphorylation and activation. A) Confocal fluorescence images of p^T14, Y15^CDK1, and DAPI in WT and *Pabpn1*‐null oocytes at GV 0–1 h, 1–2, and 2–3 h stages. Scale bar, 20 µm. B) Quantification of p^T14, Y15^CDK1 (A). Error bars, SEM. ^***^p < 0.001 by two‐tailed Student's *t‐*tests. The number of oocytes analyzed is shown (n). C) Immunofluorescence results showing p^T161^CDK1 and DAPI staining in WT and *Pabpn1*‐null oocytes at GV 0–1, 1–2, and 2–3 h stages. Scale bar, 20 µm. D) Quantification of p^T161^CDK1 (C). Error bars, SEM. ^***^p < 0.001 by two‐tailed Student's *t‐*tests. n: number of oocytes. E) Confocal fluorescence images of p^S22^Lamin A/C (p^S22^L‐A/C) and DAPI in WT and *Pabpn1*‐null oocytes at GV and GV 1–2 h stages. Scale bar, 20 µm. F) Quantification of p^S22^L‐A/C cells. Error bars, SEM. ^***^p < 0.001 by two‐tailed Student's *t‐*tests. n.s.: non‐significant. The number of oocytes analyzed is shown (n). (E). n: number of oocytes. G) Western blot results showing the protein expression levels of PABPN1, p^T14, Y15^CDK1, p^T161^CDK1, CDK1, Cyclin B1, CDC25B, and MOS in WT and *Pabpn1*‐null oocytes. DDB1 served as the loading control. The total protein from 100 oocytes was loaded into each lane. H) Diagram showing the overexpression of p^T14A, Y15F^CDK1 in WT and *Pabpn1*‐null GV oocytes. Western blot results show the protein expression levels of Flag‐p^T14A, Y15F^CDK1 in oocytes. DDB1 served as the loading control. The total protein from 100 oocytes was loaded into each lane. I) Rates of GVBD. Error bars, SEM. ^***^p < 0.001 by two‐tailed Student's *t‐*tests. n.s.: non‐significant. The number of oocytes analyzed is shown (n).

Dephosphorylation at Thr‐14 and Tyr‐15 of CDK1 (the catalytic subunit of MPF) and phosphorylation at Thr‐161 are essential for cell cycle entry into the M phase.^[^ ^24^ ^]^ p^T14, Y15^CDK1 formed nuclear puncta in GV oocytes, underwent a constant decrease, and eventually disappeared during the GVBD (**Figure** **3**A,B). However, the *Pabpn1*‐null oocytes retained high levels of p^T14, Y15^CDK1 (Figure 3A,B). The level of p^T161^CDK1 was increased in WT oocytes before GVBD, but not in *Pabpn1*‐deficient oocytes (Figure 3C,D). These observations indicate that CDK1 failed to transition from an inhibitory to an active state in *Pabpn1*‐null oocytes.

Lamin A/C is a direct substrate of CDK1.^[^ ^25^ ^]^ CDK1 phosphorylates Lamin A/C at Ser22 (p^S22^Lamin A/C), resulting in nuclear envelope disassembly.^[^ ^26^ ^]^ p^S22^Lamin A/C levels were high in WT oocytes before GVBD, but failed to accumulate in *Pabpn1*‐null oocytes (Figure 3E,F). These observations indicate that failure of CDK1 activation could not induce Lamin disassembly, resulting in GV‐stage arrest in *Pabpn1*‐null oocytes.

High levels of MPF, Mitogen‐Activated Protein Kinase (MAPK), and Moloney sarcoma oncogene (MOS) and low levels of Cyclic adenosine monophosphate (cAMP) are essential for oocytes to resume meiosis I.^[^ ^27^, ^28^, ^29^ ^]^ The dephosphorylation of CDK1 is regulated by CDC25 phosphatase and WEE1/2 kinase.^[^ ^31^ ^]^ CDC25 has three homologous proteins, CDC25A, CDC25B, and CDC25C, of which CDC25B is essential for oocyte meiotic reentry.^[^ ^32^ ^]^ Transcript levels of *Cdc25b* were significantly lower in *Pabpn1*‐deficient oocytes than in WT oocytes, whereas *Cdc25a* and *Cdc25c* transcript levels remained unchanged (Figure S3A, Supporting Information). The protein levels of CDC25B were decreased in *Pabpn1*‐null oocytes than in WT oocytes (Figure 3G). Our qRT‐PCR results showed that *Wee1/2* abundance did not change significantly in WT and *Pabpn1*‐depleted oocytes (Figure S3B, Supporting Information). Phosphodiesterase 3A (*Pde3a*) knockout oocytes are arrested at the GV stage.^[^ ^33^, ^34^ ^]^ PDE3A hydrolyzes cAMP, resulting in decreased intracellular cAMP levels, leading to oocyte meiotic reentry.^[^ ^34^ ^]^ The transcript levels of *Pde3a* remained unchanged in the WT and *Pabpn1*‐depleted oocytes (Figure S3C, Supporting Information). These observations indicate that low CDC25B levels lead to inhibition of p^T14, Y15^CDK1 dephosphorylation, resulting in low phosphorylation of Lamin A/C and defects in GVBD.

Western blotting showed a significant decrease in the levels of p^T161^CDK1, CDC25B, and MOS proteins in PABPN1‐deficient oocytes. However, the levels of p^T14, Y15^CDK1 remained stable. (Figure 3G). Overexpression of the activated forms of CDK1 (p^T14A, Y15F^CDK1) in oocytes lacking *Pabpn1* (Figure 3H) resumed meiosis I, and ≈70% of the oocytes underwent GVBD (Figure 3I). The above experimental results indicate that *Pabpn1*‐null oocytes could not resume meiotic processes because of insufficient p^T14, Y15^CDK1 dephosphorylation.

### 2.4 Knockout of *Pabpn1* Affects Translation of Cytoplasmic Transcripts in Oocytes

**Figure 4:**
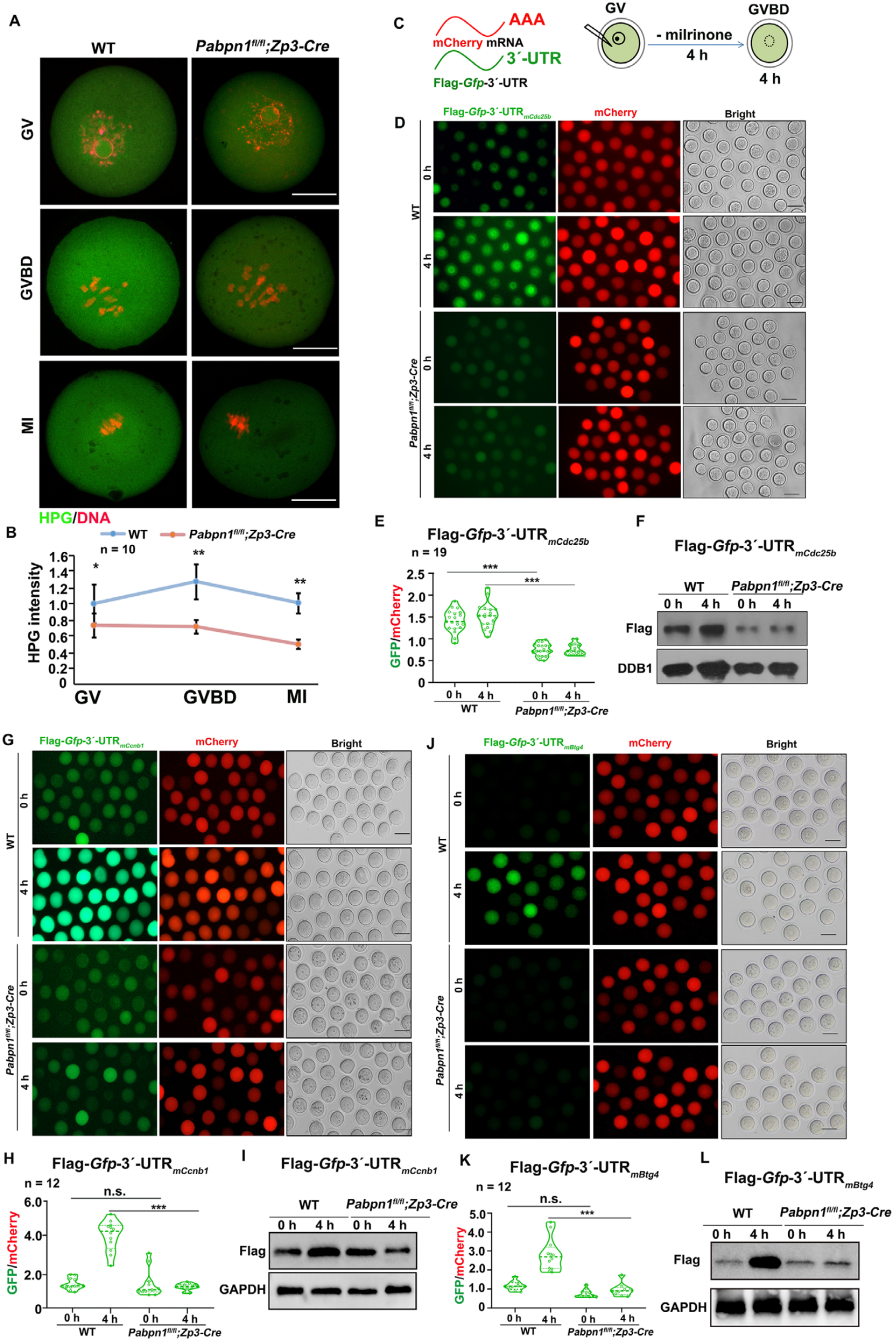
Translation analysis of the WT and *Pabpn1‐*null oocytes. A) HPG fluorescence staining results showing protein synthesis in WT and *Pabpn1^fl/fl^;Zp3‐Cre* mouse oocytes. Scale bars, 20 µm. Green represents HPG, and red represents DNA. B) Quantification of HPG signal intensity in (A). Error bars, SEM. ^*^p < 0.05, ^**^p < 0.01 by two‐tailed Student's *t‐*tests. n: number of oocytes. C) Illustration of mRNA microinjection into WT and *Pabpn1‐*null GV oocytes. D–F) Fluorescence microscopy (D), quantification (E), and western (F) results illustrate the expression of Flag‐GFP fused with *mCdc25b* 3′‐UTR and mCherry. Scale bar, 100 µm. The number of oocytes analyzed at each stage is indicated. Error bars indicate SEM. ^***^ *p* < 0.001 by two‐tailed Student's *t‐*tests. G–I) Fluorescence microscopy (G), quantification (H), and western (I) results illustrating the expression of Flag‐GFP fused with *mCcnb1* 3′‐UTR and mCherry. Scale bar, 100 µm. The number of oocytes analyzed at each stage is indicated. Error bars indicate SEM. ^***^ *p* < 0.001 by two‐tailed Student's *t‐*tests. n.s.: non‐significant. J–L) Fluorescence microscopy (J), quantification (K), and western blot (L) results illustrating the expression of Flag‐GFP fused with *mBtg4* 3′‐UTR and mCherry. Scale bar, 100 µm. The number of oocytes analyzed at each stage is indicated. Error bars indicate SEM. ^***^ *p* < 0.001 by two‐tailed Student's *t‐*tests. n.s.: non‐significant.

The translation level of WT oocytes gradually increased during meiotic maturation, whereas that of *Pabpn1*‐null oocytes decreased significantly compared to that of WT oocytes during meiotic maturation (**Figure** **4**A,B). Next, we constructed mCherry and Flag‐*Gfp‐*3′‐UTR*_mCdc25b_* transcripts to further verify the translational levels of these transcripts in WT and *Pabpn1*‐null oocytes (Figure 4C,D). The expression of Flag‐GFP and mCherry proteins was detected in oocytes by epifluorescence and quantified using ImageJ software (Figure 4D,E). The translational activity of Flag‐GFP was lower in the *Pabpn1*‐null oocytes than in the WT oocytes (Figure 4D–F). Similar to the 3′‐UTR*_mCdc25b_* transcript, the translation activity of Flag‐*Gfp‐*3′‐UTR*_mCcnb1_* (Figure 4G–I) and Flag‐*Gfp‐*3′‐UTR*_mBtg4_* (Figure 4J–L) was lower in *Pabpn1*‐null oocytes than in WT oocytes. These results indicate that the deletion of *Pabpn1* affects the translational activity of transcripts related to MPF and deadenylation factors.

### 2.5 PABPN1 Promotes Meiotic Resumption of Fully Grown GV Oocytes

**Figure 5:**
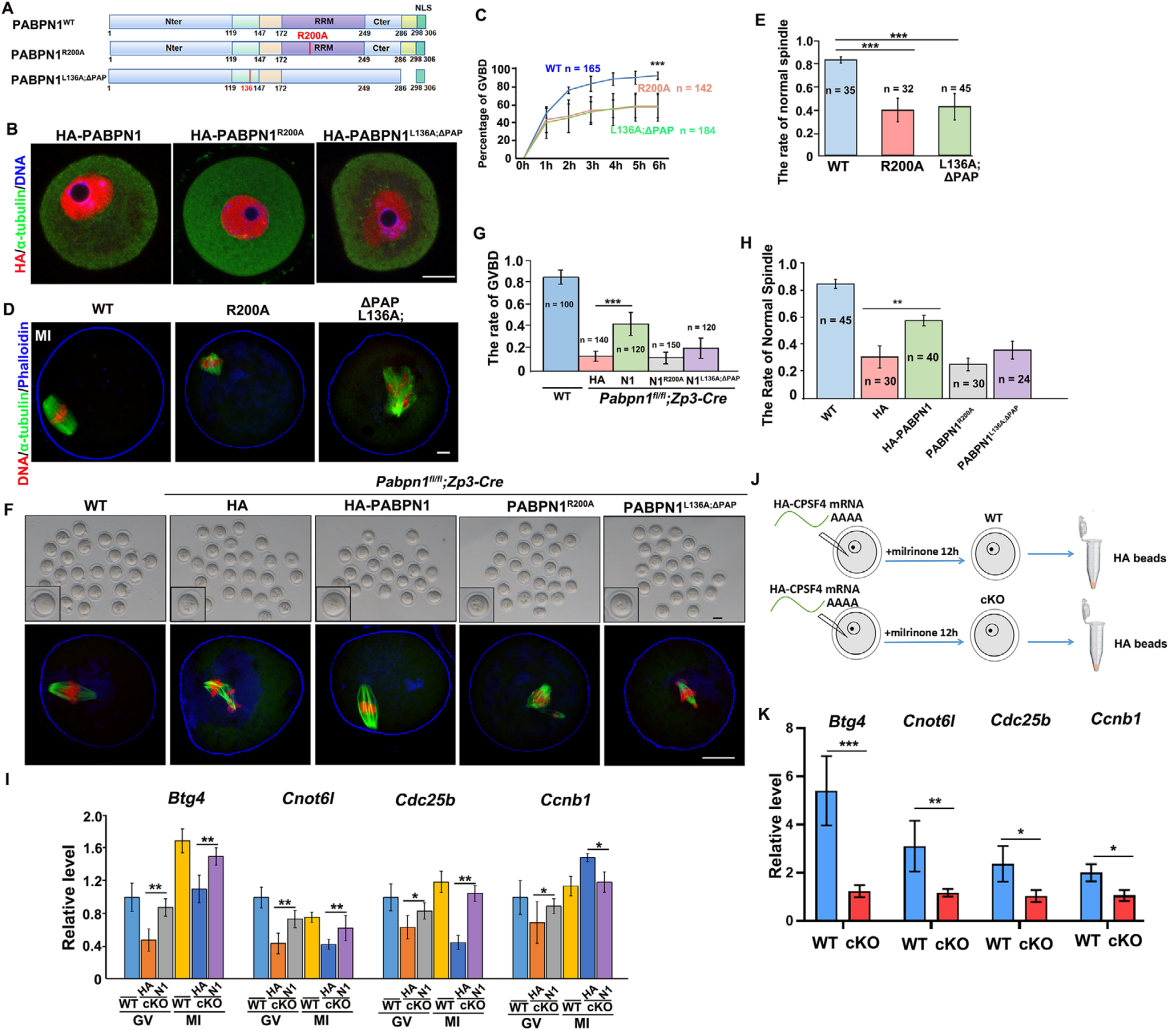
Different domains of PABPN1 affect the meiotic maturation of oocytes. A) Diagram showing the domains of the PABPN1. Schematic diagrams showing three constructs: PABPN1 full‐length (WT), RRM mutation (R200A, disrupting RNA binding), and PAP deletion and L136 mutation (L136A;ΔPAP, disrupting binding to PAPα). B) Immunofluorescence results show expression of PABPN1 (WT), PABPN1 (R200A), and PABPN1 (L136A;ΔPAP) in oocytes. Scale bar, 20 µm. C) Percentage of patients with GVBD (D). Error bars, SEM. ^***^p < 0.001 by two‐tailed Student's *t‐*tests. n.s.: non‐significant. The number of oocytes analyzed is shown (n). D) Immunofluorescence shows spindles in oocytes overexpressing PABPN1 (WT), PABPN1 (R200A), or PABPN1 (L136A; PAP). Scale bar = 20 µm. E) Quantification of normal spindles in oocytes overexpressing the PABPN1 constructs. Error bars, SEM. ^***^p < 0.001 by two‐tailed Student's *t‐*tests. The number of oocytes analyzed is shown (n). F) Representative images of WT and *Pabpn1^fl/fl^;Zp3‐Cre* mouse oocytes showing GVBD after overexpression of HA, HA‐PABPN1, PABPN1^R200A^, and PABPN1^L136A;ΔPAP^. Scale bar, 100 µm. G) GVBD rate in (F). HA, (N1) HA‐PABPN1, (N1^R200A^) PABPN1^R200A^, and (N1^L136A;ΔPAP^) PABPN1^L136A;ΔPAP^. Error bars, SEM. ^***^p < 0.001 by two‐tailed Student's *t‐*tests. The number of oocytes analyzed is shown (n). H) Quantification of normal spindles in oocytes overexpressing WT or mutant PABPN1 (HA, HA‐PABPN1, PABPN1^R200A^, and PABPN1^L136A;ΔPAP^). Error bars, SEM. ^**^p < 0.01 by two‐tailed Student's *t‐*tests. The number of oocytes analyzed is shown (n). I) Quantitative RT‐PCR results showing the mRNA levels of specific transcripts (*Btg4, Cnot6l, Cdc25b*, and *Ccnb1*) in WT and *Pabpn1*‐null oocytes overexpressing HA and HA‐PABPN1 at GV and MI stages. Error bars, SEM. ^*^ *p* < 0.05, ^**^ *p* < 0.01 by two‐tailed Student's *t‐*tests. J) RNA immunoprecipitation assay for detecting CPSF4 binding to mRNAs in WT and *Pabpn1^fl/fl^;Zp3‐Cre* mouse oocytes. K) RT‐qPCR was used to measure the relative abundance of mRNAs immunoprecipitated by HA‐CPSF4. Error bars, SEM. ^*^p < 0.05, ^**^p < 0.01 and ^***^p < 0.001 by two‐tailed Student's *t‐*tests.

To investigate whether PABPN1 functions directly during oocyte meiosis, we constructed WT and mutant forms of PABPN1 and overexpressed them in GV oocytes to explore the effects of PABPN1 on oocyte maturation (**Figure** **5**A,B). PABPN1^R200A^ disrupted the interaction between PABPN1 and mRNA, while PABPN1^L136A;ΔPAP^ disrupted the interaction between PABPN1 and the mRNA polyadenylation enzyme PAPα (Figure S4A, Supporting Information).^[^ ^35^ ^]^

Immunofluorescence results showed that HA‐PABPN1, HA‐PABPN1^R200A^, and HA‐PABPN1^L136A;ΔPAP^ were successfully expressed in the GV oocytes (Figure 5B). Overexpression of PABPN1^R200A^ and PABPN1^L136A;ΔPAP^ both affected GVBD rates (Figure 5C). Immunofluorescence results showed that most PABPN1^R200A^ and PABPN1^136A;PAPΔ^ overexpressing GVBD oocytes had distorted spindles (Figure 5D,E). Similar to *Pabpn1*‐null oocytes, oocytes overexpressing PABPN1^R200A^ and PABPN1^L136A;ΔPAP^ exhibited GVBD defects. These results indicated that PABPN1 affects meiotic resumption during oocyte meiotic maturation.

To further confirm that PABPN1 could promote the resumption of oocytes from meiosis, we overexpressed WT and inactivated forms of PABPN1 (PABPN1^R200A^ and PABPN1^L136A;ΔPAP^) in *Pabpn1*‐null oocytes to test whether the oocytes could develop GVBD (Figure 5F). Overexpressing of the RNA‐binding‐deficient PABPN1 mutants (PABPN1^R200A^and PABPN1^L136A;ΔPAP^) in cKO oocytes failed to rescue the GVBD rate, which remained indistinguishable from that of cKO oocytes. This result highlights that the RNA‐binding activity of PABPN1 is essential for its function in promoting meiotic resumption, as the mutants (defective in poly(A)‐binding) could not restore GVBD efficiency despite being overexpressed. In *Pabpn1*‐null oocytes, spindle morphology was significantly improved by PABPN1 overexpression, but not by PABPN1^R200A^ and PABPN1^L136A;ΔPAP^ (Figure 5F–H). Therefore, PABPN1 is essential for the development of GVBD and the maintenance of spindle morphology in oocytes.

We collected GV and MI oocytes from WT and *Pabpn1* knockout mice, and rescued the *Pabpn1* knockout oocytes by expressing full‐length PABPN1. Because polyadenylation adds a poly(A) tail to the 3′ end of mRNA, enhancing its stability, extending its lifespan, and preventing rapid degradation, we evaluated the transcript levels of the MPF factors (*Cdc25b* and *Ccnb1*) and deadenylation factors (*Btg4* and *Cnot6l*). Our findings revealed that *the Pabpn1* knockout significantly reduced the transcript levels of these factors compared to those in WT oocytes. However, the restoration of full‐length PABPN1 partially recovered these transcript levels (Figure 5I). Regarding the restoration of transcript levels, we acknowledge that transcription is absent in fully grown oocytes. Therefore, the observed recovery is likely due to the stabilization of existing transcripts through polyadenylation rather than new transcription. Thus, PABPN1 likely influences the polyadenylation of these transcripts, affecting both transcript and translation levels.

To investigate whether PABPN1 collaborates with the cytoplasmic polyadenylation factor CPSF4 to promote polyadenylation of downstream target genes, we performed RNA immunoprecipitation (RIP) experiments to examine the binding of CPSF4 to PABPN1 target genes (*Cdc25b*, *Ccnb1, Btg4*, and *Cnot6l*) in WT and *Pabpn1*‐null oocytes (Figure 5J). The RIP results revealed that in *Pabpn1*‐null oocytes, the binding of CPSF4 to PABPN1 target genes was significantly reduced (Figure 5K). These results indicate that PABPN1 and CPSF4 cooperate in the oocyte cytoplasm to drive polyadenylation of downstream target genes, which is a key process for GVBD and spindle formation.

### 2.6 Knockout of *Pabpn1* Affects poly(A) Tail Lengths of Transcripts in Oocytes

**Figure 6:**
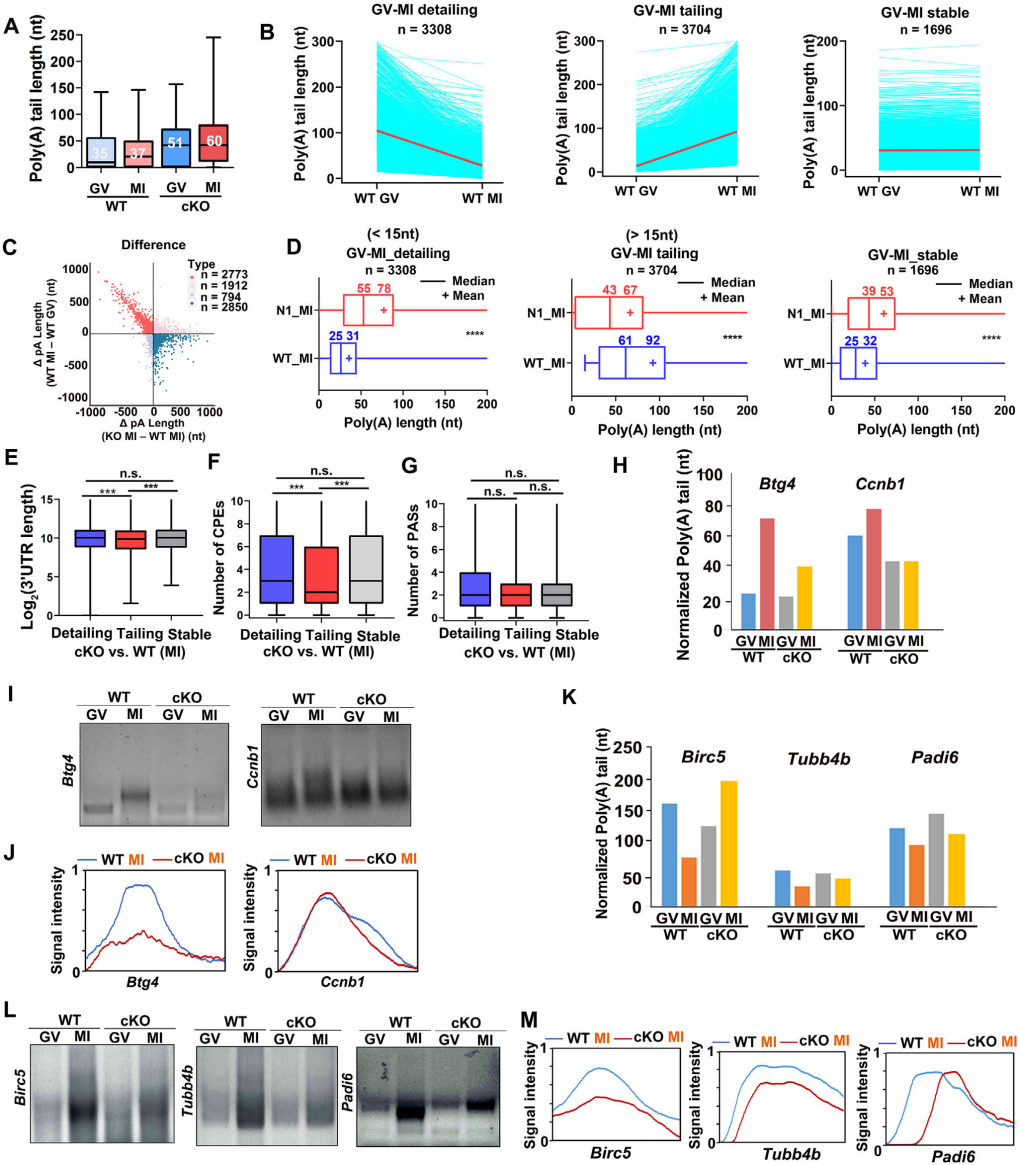
PABPN1 deficiency leads to shortened poly(A) tails of polyadenylated transcripts during the transition from the GV to the MI stage. A) Boxplot showing the distribution of poly(A) transcript lengths in WT, *Pabpn1*‐null GV, and MI oocytes. B) The line graph illustrates the changes in poly(A) tail length of transcripts during the transition from the GV to MI stage under normal conditions. C) Scatter plot comparing poly(A) length changes between WT and *Pabpn1*‐null MI oocytes (KO/WT) and the WT pattern (MI oocyte/GV). Each dot represents a gene, and the colors group the genes according to the poly(A) change tendency: detailing in the WT pattern but tailing in KO MI oocytes (red), and tailing in the WT pattern but detailing in KO MI oocytes (blue). (n) Number of genes in each group. D) Boxplot showing the distribution of poly(A) tail lengths in WT and *Pabpn1*‐null MI‐stage oocytes from transcripts that undergo detailing, tailing, or maintain stable poly(A) tail lengths during the normal GV‐MI transition. E–G) Average 3ˊ‐UTR length (E), number of CPEs (F), and PASs (G) in the 3ˊ‐UTR of transcripts detail, tail, and stability at MI stages in *Pabpn1^fl/fl^;Zp3‐Cre* oocytes compared with WT oocytes. Error bars, SEM. ^***^p < 0.001 by two‐tailed Student's *t‐*tests. n.s.: non‐significant. H) Poly (A) tail lengths of specific transcripts in WT and *Pabpn1*‐deficient oocytes. I) Variations in the poly(A) tail length of transcripts from WT and *Pabpn1^fl/fl^;Zp3‐Cre* mouse oocytes were evaluated using poly(A) tail assays. J) Relative signal intensity (*y*‐axis) and PCR product length according to mobility (*x*‐axis) (I). K) In WT and *Pabpn1*‐deficient oocytes, the lengths of the poly(A) tails of specific transcripts. L) Poly(A) tail length differences in transcripts from WT and *Pabpn1^fl/fl^;Zp3‐Cre* mouse oocytes were measured using poly(A) tail assay. M) Signal intensity (*y*‐axis) and PCR product size based on mobility (*x*‐axis), as shown in (L).

To investigate whether PABPN1 affects oocyte meiotic maturation by remodeling the transcript poly(A) tail length, we adapted a previously reported method and performed PAIso‐seq on WT and *Pabpn1*‐null oocytes at the GV and MI stages^[^ ^36^, ^37^ ^]^ (Figure S4B, Supporting Information). At both stages, the overall poly(A) tail length in the *Pabpn1*‐null oocytes was longer than that in the WT oocytes (**Figure** **6**A). We further classified the transcripts into three groups based on the changes in poly (A) tail length from the GV to MI stages: GV‐MI tailing for transcripts with elongated poly(A) tails, GV‐MI detailing for those with shortened tails, and GV‐MI stability for the remaining transcripts (Figure 6B).

Further analysis using a four‐quadrant plot revealed that transcripts undergoing tailing from the GV to the MI stage in WT oocytes showed a marked reduction in poly(A) tail length in *Pabpn1*‐null MI oocytes (Figure 6C). Sequence analysis demonstrated a significant shortening of the poly(A) tails in GV‐MI tailing transcripts after *Pabpn1* knockout, with a median length of 61 nt reduced to 43 nt (Figure 6D). However, stable GV‐MI and detailing transcripts in WT oocytes displayed significantly longer poly(A) tails in the *Pabpn1*‐null MI oocytes at the same stage. Collectively, these findings suggest that PABPN1 specifically regulates the polyadenylation of transcripts undergoing tailing during the GV‐MI transition.

The length of the transcript's 3′‐UTR and its regulatory elements can influence poly(A) tail length. Further analysis showed that transcripts with elongated poly(A) tails in *Pabpn1*‐null MI oocytes had shorter 3′‐UTRs and fewer cytoplasmic polyadenylation elements (CPEs) compared to WT oocytes (Figure 6E,F), whereas the number of polyadenylation signals (PASs) remained unchanged (Figure 6G). In *Pabpn1*‐null oocytes, numerous transcripts exhibited extended poly(A) tails compared to those in WT oocytes at the same developmental stage. Further investigations were conducted to explore whether *Pabpn1* directly regulates polyadenylation of key factors involved in maternal mRNA degradation, such as *Btg4* and *Cnot6l*.

Analysis of PAIso‐seq results revealed that numerous transcripts (*Btg4*, and *Ccnb1*) involved in oocyte meiosis exhibited poly(A) tail elongation from GV to MI oocytes, whereas, in *Pabpn1*‐knockout oocytes, the poly(A) tails were shortened (Figure 6H). Furthermore, poly(A) tail assays confirmed these transcripts had significantly extended poly(A) tails in WT oocytes, whereas these tails were shortened in *Pabpn1*‐null oocytes (Figure 6I,J). These findings indicated that PABPN1 likely regulates oocyte meiotic maturation by modulating the poly(A) tail length of the transcripts. Moreover, the PAT assay analysis of the downstream target genes (*Birc5, Tubb4b*, and *Padi6*) of *Btg4* and *Cnot6l* revealed that the poly(A) tail lengths of these genes were significantly extended in *Pabpn1*‐null oocytes (Figure 6K–M).

These results suggest that PABPN1 promotes the meiotic maturation of oocytes through a dual regulatory mechanism; on the one hand, it directly facilitates poly(A) tail elongation and translation of key MPF components; however, it regulates poly(A) tail length and expression of key maternal mRNA degradation factors, such as *Btg4*.

### 2.7 Maternal Transcripts are Abnormal in *Pabpn1‐*null Oocytes

**Figure 7:**
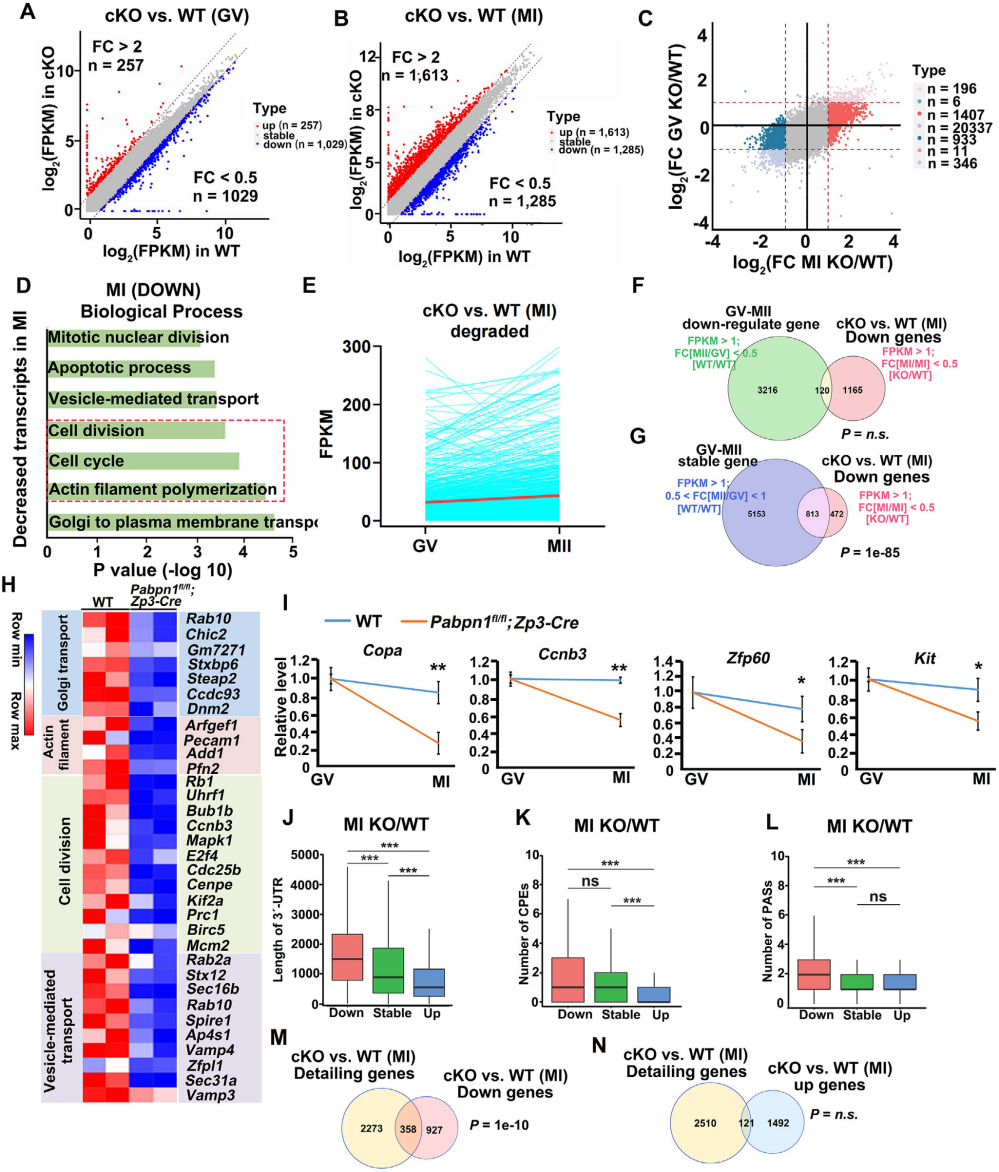
Analysis of decreased transcripts in WT and *Pabpn1*‐deleted oocytes at the MI stage. A,B) A scatter plot depicting relative transcript levels in WT and *Pabpn1*‐null oocytes at the GV and MI stages. Transcripts (n) with more than a 2‐folds increase or decrease are marked in red and blue, respectively. FC: fold change. C) Scatter plot comparing transcript levels between WT, *Pabpn1*‐null GV (KO/WT), and MI oocytes (KO/WT). Each dot represents a gene and the colors group the genes according to their transcript levels. (n) Number of genes in each group. D) Gene Ontology analysis of transcripts whose levels were decreased in MI oocytes from *Pabpn1^fl/fl^;Zp3‐Cre* mice compared to those in WT mice. E) The line graph illustrates the changes in transcripts that decreased at the MI stage in *Pabpn1*‐deficient oocytes compared with those in WT oocytes, showing their variation in normal GV‐MII oocytes. F) The Venn diagram illustrates transcripts whose levels were decreased in *Pabpn1^fl/fl^;Zp3‐Cre* mice compared to WT mice at the MI stage, along with transcripts that were downregulated during the WT GV‐MII transition. G) The Venn diagram illustrates transcripts whose levels decreased in *Pabpn1^fl/fl^;Zp3‐Cre* mice compared to those in WT mice at the MI stage, along with transcripts that were stable during the WT GV‐MII transition. H) A heatmap depicting the expression profiles of transcripts with reduced levels at the MI stage in *Pabpn1^fl/fl^;Zp3‐Cre* mice compared to WT controls. I) RT‐qPCR results showing the relative mRNA levels of selected transcripts in WT and *Pabpn1^fl/fl^;Zp3‐Cre* mouse oocytes at different time points. Error bars, s.e.m. ^*^p < 0.05, ^**^p < 0.01, two‐tailed Student's *t*‐test. (J‐L) Average 3ˊ‐UTR length J) and numbers of CPEs K) and PASs L) in the 3ˊ‐UTR of transcripts decreased, stable, and increased at MI stages in *Pabpn1^fl/fl^;Zp3‐Cre* oocytes compared with the WT oocytes. ^***^p < 0.001 by two‐tailed Student's *t*‐test. n.s.: non‐significant. M) Venn diagram illustrating the intersection of transcripts with shortened poly(A) tails in *Pabpn1*‐deficient oocytes compared to WT oocytes at the MI stage, and transcripts that decreased during this period. N) The Venn diagram highlights the overlap between transcripts with shortened poly(A) tails in *Pabpn1*‐deficient oocytes and those that increased at the MI stage compared to WT oocytes.

PABPN1 is an RNA‐binding protein that associates with poly(A) tails, influencing their length and potentially altering transcript levels within oocytes. To explore this, we performed transcriptome sequencing on WT and *Pabpn1*‐knockout oocytes at the GV and MI stages. The replicates displayed a high correlation coefficient (Figure S4C, Supporting Information). At the GV stage, 257 genes were upregulated (FC > 2), and 1029 genes were downregulated (FC < 0.5) in *Pabpn1*‐null oocytes compared to WT oocytes (**Figure** **7**A). At the MI stage, the number of upregulated (1613) and downregulated (1285) transcripts was significantly higher in *Pabpn1*‐null oocytes than in their WT counterparts (Figure 7B).

Further analysis using a four‐quadrant plot revealed a significant overlap among transcripts that decreased at both GV and MI stages (Figure 7C). Gene Ontology (GO) analysis of the transcripts decreased during the MI stage, indicating their involvement in processes such as Golgi‐to‐plasma membrane transport, actin filament polymerization, cell cycle, cell division, and vesicle‐mediated transport (Figure 7D). The transcripts decreased in the MI stage following *Pabpn1* deficiency and were stable from the normal GV‐to‐MII stages (Figure 7E). A Venn diagram further revealed that 120 transcripts downregulated following *Pabpn1* knockout also decreased during the normal GV‐to‐MII transition, whereas 813 transcripts remained stable throughout this transition (Figure 7F,G). These findings suggest that *Pabpn1* knockout leads to a pronounced reduction in transcripts that are typically stable throughout the GV‐to‐MII stages.

Consistent with the transcriptome sequencing results, heatmap and RT‐qPCR analyses showed that several transcripts (*Copa, Zfp60, Ccnb3*, and *Kit*) remained stable in maturing oocytes but were destabilized in *Pabpn1*‐knockout oocytes (Figure 7H,I). Further analysis of transcripts downregulated in MI‐stage *Pabpn1*‐knockout oocytes than in WT oocytes, revealing these transcripts had longer 3′‐UTRs than those that were increased or stable (Figure 7J). Moreover, the 3′‐UTRs of the decreased transcripts contained more CPEs and PASs than the increased transcripts following *Pabpn1* knockout (Figure 7K,L). Collectively, these findings indicate that *Pabpn1* deletion leads to the destabilization of numerous transcripts by disrupting their polyadenylation.

In *Pabpn1*‐null MI oocytes, compared with WT oocytes at the same stage, 358 transcripts with shortened poly(A) tails exhibited reduced levels (Figure 7M), whereas only 121 transcripts showed increased expression (Figure 7N). Therefore, PABPN1 likely regulates transcript levels by modulating polyadenylation.

**Figure 8:**
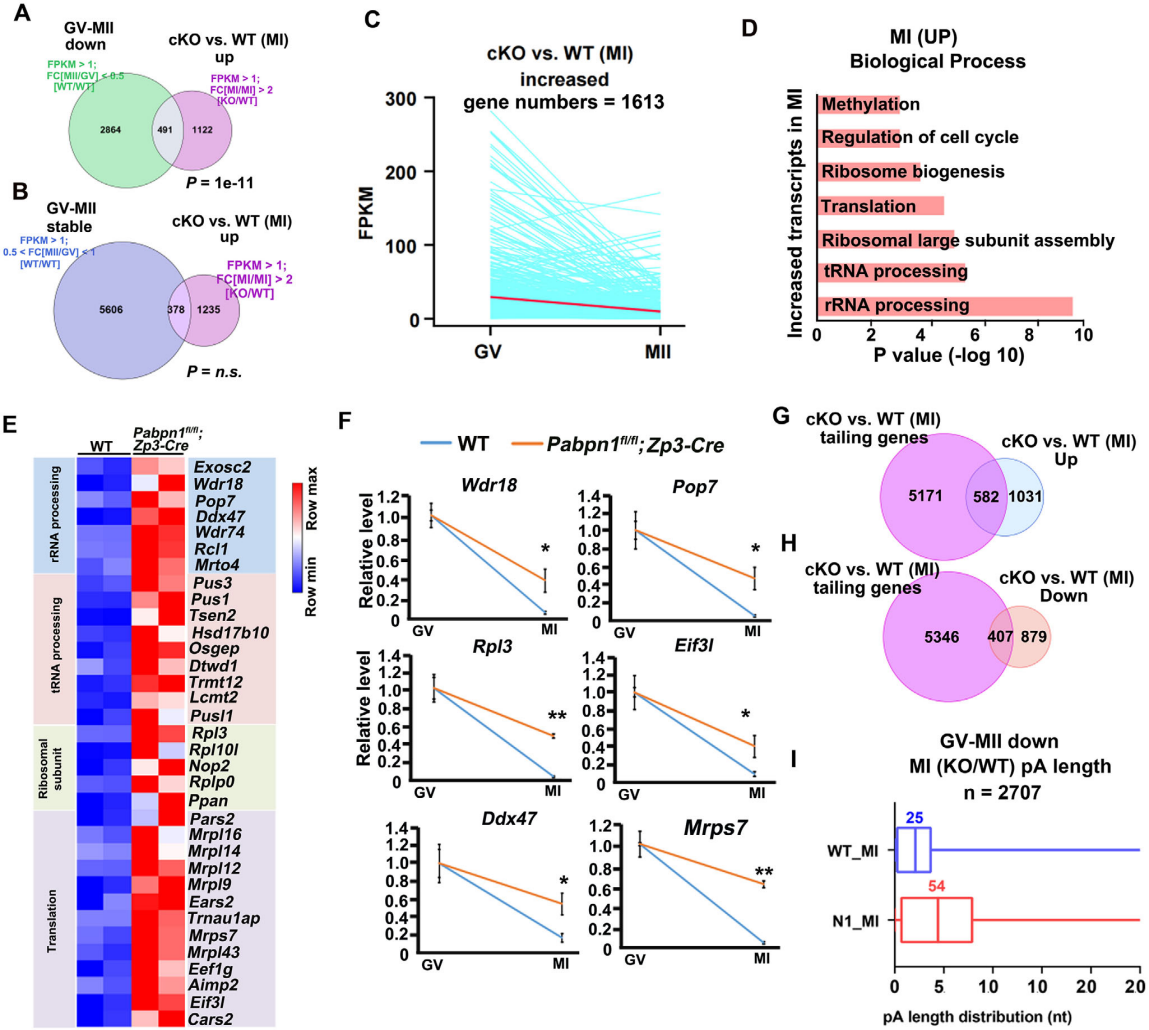
Analysis of increased transcripts in WT and *Pabpn1*‐deleted oocytes at the MI stage. A) The Venn diagram illustrates transcripts whose levels were increased in *Pabpn1^fl/fl^;Zp3‐Cre* mice compared to those in WT mice at the MI stage, along with transcripts that were downregulated during the WT GV‐MII transition. B) The Venn diagram illustrates transcripts whose levels were increased in *Pabpn1^fl/fl^;Zp3‐Cre* mice compared to those in WT mice at the MI stage, along with transcripts that were stable during the WT GV‐MII transition. C) The line graph illustrates the changes in transcripts that increased at the MI stage in *Pabpn1*‐deficient oocytes compared with WT oocytes, showing their variation in normal GV‐MII oocytes. D) GO analysis of transcripts showing elevated levels in MI oocytes from *Pabpn1^fl/fl^;Zp3‐Cre* mice compared to those in WT mice. E) Heatmap depicting the expression patterns of transcripts with elevated levels at MI stage in *Pabpn1^fl/fl^;Zp3‐Cre* mice relative to WT mice. F) RT‐qPCR analysis of mRNA levels for selected transcripts in WT and *Pabpn1^fl/fl^;Zp3‐Cre* mouse oocytes at different time points. Data are presented as mean ± SEM. ^**^p < 0.01 (two‐tailed Student's *t*‐test); n.s., non‐significant. G) Venn diagram illustrating the overlap between transcripts with extended poly(A) tails in *Pabpn1*‐deficient oocytes and those that increased at the MI stage compared with WT oocytes. H) Venn diagram illustrating the overlap between transcripts with extended poly(A) tails in *Pabpn1*‐deficient oocytes and those that decreased at the MI stage compared with WT oocytes. (I) Boxplot showing the distribution of poly(A) tail lengths in transcripts that normally decrease during the GV‐MII transition in *Pabpn1*‐deficient and WT MI oocytes.

Next, we analyzed the transcripts that increased during meiosis following the *Pabpn1* knockout. A Venn diagram revealed that among the transcripts that increased in the MI stage following *Pabpn1* knockout, 491 transcripts decreased during the normal GV‐MII transition (**Figure** **8**A), whereas 378 transcripts remained stable (Figure 8B). Further analysis showed these upregulated transcripts typically exhibited decreased levels from the GV to the MII stage under normal conditions (Figure 8C). Many of the upregulated transcripts in *Pabpn1*‐null oocytes were associated with genes involved in rRNA processing, tRNA processing, and translation (Figure 8D,E). qRT‐PCR validation confirmed that transcripts normally decreased during oocyte maturation; for example, *Wdr18, Pop7, Ddx47, Rpl3, Eif3l*, and *Mrps7* were partially stabilized in *Pabpn1*‐null MI oocytes (Figure 8F). These findings indicate that *Pabpn1* knockout disrupts the normal degradation of maternal transcripts during oocyte maturation.

In *Pabpn1*‐null MI oocytes, compared with WT oocytes at the same stage, 582 transcripts with elongated poly(A) tails exhibited increased levels (Figure 8G), whereas 407 transcripts exhibited decreased expression (Figure 8H). Further analysis of transcripts that decreased during the GV‐to‐MII transition revealed that *Pabpn1* deficiency in MI oocytes resulted in general elongation of poly(A) tails compared to WT oocytes (Figure 8I). During meiosis, PABPN1 promotes polyadenylation and stabilization of deadenylation factors, facilitating deadenylation of maternal target genes and reducing transcript levels.

## 3 Discussion

In our previous study, PABPN1 colocalizes with the splicing factor CPSF4 in the NPAD region of growing oocyte nuclei, where it regulates mRNA processing and storage. As oocyte meiosis resumes and germinal vesicles break down, PABPN1 is dispersed into the cytoplasm. In this study, we identified the functional role of PABPN1 in the cytoplasm, where it promotes polyadenylation and stability of specific maternal transcripts. PABPN1 regulates MPF‐related factors and the maternal mRNA degradation factor *Btg4*, coupling the polyadenylation of maternal transcripts with the degradation and progression of oocyte meiosis.

In this study, we collected *Pabpn1*‐null oocytes and found that most failed to undergo GVBD. Among the oocytes that underwent GVBD, we observed severe spindle abnormalities that ultimately led to fertilization failure (Figures 1, 2). Further analysis revealed that the GVBD defect in *Pabpn1*‐null oocytes was caused by MPF inactivation. Overexpression of the dephosphorylated activated forms of p^T14A, Y15F^CDK1 restored GVBD in 70% of the *Pabpn1*‐null oocytes (Figure 3). The GVBD defects observed in *Pabpn1*‐null oocytes may be partially attributed to the abnormal storage and processing of maternal transcripts during oocyte growth. To eliminate potential effects during the growth phase, we constructed mutant forms of PABPN1 (PABPN1^R200A^ and PABPN1^L136A;ΔPAP^) that lack both functional domains with mRNA and PAPα. Overexpression of PABPN1^R200A^ and PABPN1^L136A;ΔPAP^ in WT GV‐stage oocytes resulted in phenotypes similar to those observed in *Pabpn1*‐null oocytes, including a failure to undergo GVBD and spindle abnormalities (Figure 5). In addition, restoration of WT PABPN1 in *Pabpn1*‐null oocytes partially rescued the GVBD and spindle defects. These results confirm that PABPN1 functions in the cytoplasm and that its RRM and PAP domains are crucial for its activity in maturing oocytes.

Next, we performed PAIso‐seq and transcriptome sequencing of both WT and *Pabpn1*‐null oocytes to investigate the effects of PABPN1 deficiency on oocyte meiosis. The PAIso‐seq results showed that PABPN1 directly influenced *Ccnb1* polyadenylation. In addition, PABPN1 affected polyadenylation of the maternal mRNA decay factor *Btg4* (Figure 6). Transcriptome sequencing revealed that *Pabpn1*‐null MI oocytes contained numerous transcripts that failed to degrade properly (Figure 8). We confirmed that PABPN1 directly influences the poly(A) tails of *Ccnb1* and *Btg4*. In addition, 3′‐UTR reporter assays demonstrated that *Pabpn1* deficiency decreased the translation of these proteins (Figure 4).

Our findings reveal that PABPN1 orchestrates oocyte meiotic progression through dual regulatory mechanisms involving *Ccnb1/Cdc25b* (key components of the maturation‐promoting factor, MPF) and *Btg4/Cnot6/6l* (deadenylation mediators). Cyclin B1 (encoded by *Ccnb1*) and CDC25B are critical for CDK1 activation, a master driver of germinal vesicle breakdown (GVBD) and spindle assembly. PABPN1‐mediated polyadenylation stabilizes *Ccnb1* and *Cdc25b* transcripts, ensuring their timely translation to sustain CDK1 activity. Conversely, PABPN1 interacts with BTG4 and CNOT6/6L, core components of the CCR4‐NOT deadenylase complex, to promote targeted deadenylation and degradation of maternal mRNAs. This dual role allows PABPN1 to dynamically balance the accumulation and clearance of transcripts essential for meiosis.

In somatic cells, PABPN1 regulates polyadenylation and poly(A) tail length in the nucleus with the help of PAPα and CPSF4, whereas PABPC1 governs translation and mRNA stability in the cytoplasm.^[^ ^38^ ^]^ However, in this study, PABPN1 promoted polyadenylation of cytoplasmic transcripts in oocytes. Previous studies have reported that both CPSF4 and PAPα promote cytoplasmic polyadenylation, and their deletion causes meiotic defects in oocytes.^[^ ^16^, ^17^ ^]^ In this study, based on the interactions between PABPN1, PAPα, and CPSF4, we hypothesized that PABPN1 may interact with PAPα and CPSF4 to promote cytoplasmic polyadenylation. To further validate this hypothesis, it would be valuable to knock down PAPα and CPSF4, and perform PAIso‐seq sequencing to determine whether their target genes overlap with those of PABPN1. In addition, endogenous mass spectrometry analysis of PABPN1 following germinal vesicle breakdown in oocytes is required to identify the PABPN1‐interacting proteins in the cytoplasm. Thus, PABPN1 might participate in polyadenylation in collaboration with other proteins. To investigate the maternal transcripts by which PABPN1 promotes polyadenylation, an RIP experiment for PABPN1 was performed after oocyte meiosis to directly identify maternal transcripts targeted by PABPN1.

In conclusion, this study enhances our understanding of the molecular mechanisms governing oocyte maturation by elucidating how PABPN1 regulates polyadenylation of *Ccnb1* and *Btg4* to facilitate oocyte meiotic progression. Our findings offer a more comprehensive view of the regulatory networks involved in maternal transcript stability and degradation and highlight the importance of polyadenylation in maintaining the integrity of oocyte maturation. Future studies should focus on elucidating the interaction network of PABPN1 in the cytoplasm and exploring the collaboration between PABPN1, PAPα, and CPSF4.

## 4 Experimental Section

### Animals

The *Pabpn1^fl/fl^* mouse strain was generated through CRISPR‐Cas9‐based gene targeting, with all mice maintained on a C57BL/6J genetic background. Animal experiments were performed in strict compliance with the guidelines and regulations of Zhejiang University, and the study protocol (ZJU20210252) received approval from the University's Institutional Animal Care and Use Committee.

### Histological and Immunofluorescent Analyses

After dehydration, ovaries were paraffin‐embedded, sectioned, deparaffinized in xylene (3 × 5 min), rehydrated in absolute and 95% ethanol (5 min each), and stained with H&E.

For immunohistochemical analyses, the sections were incubated in 3% H_2_O_2_ for 10 min, cooled to room‐temperature after boiling in 10 mm sodium citrate buffer (pH 6.0) for 15 min, washed, blocked with 10% donkey serum for 60 min, and incubated with primary antibodies overnight at 4 °C. The slides were then washed and incubated with biotin‐labeled secondary antibodies. The antibodies were detected using the VECTASTAIN ABC Kit and 3,3′‐diaminobenzidine peroxidase substrate Kit (Vector Laboratories, Burlingame, CA, USA). Slides were counterstained with hematoxylin and eosin and imaged using an 80i microscope equipped with a camera (Nikon, Tokyo, Japan).

For immunohistochemistry, sections were treated with 3% H_2_O_2_ for 10 min, subjected to antigen retrieval in 10 mm sodium citrate buffer (pH 6.0) at boiling for 15 min, cooled, washed, and blocked with 10% donkey serum for 1 h. They were then incubated with primary antibodies overnight at 4 °C, followed by washing and incubation with biotin‐labeled secondary antibodies. Detection was performed using the VECTASTAIN ABC Kit and 3,3′‐diaminobenzidine (DAB) peroxidase substrate kit (Vector Laboratories, Burlingame, CA, USA). Slides were counterstained with H&E and imaged with a Nikon 80i microscope (Tokyo, Japan).

### Microinjection of Oocytes

Fully grown oocytes were prepared for microinjection by incubation in M2 medium (M7167; Sigma–Aldrich, St. Louis, MO, USA) supplemented with 2 µm milrinone. Using an Eppendorf TransferMan NK2 micromanipulator, 5–10 pl of RNA sample (500 ng µL^−1^) was injected into each oocyte. Post‐injection, oocytes were washed and cultured in M16 medium (M7292; Sigma–Aldrich) at 37 °C under 5% CO_2_.

### Oocyte Culture

Four‐week‐old female mice were administered 5 IU of PMSG, and oocytes were collected 46–48 h in M2 medium. The oocytes were then cultured in M16 medium micro drops under mineral oil (M5310; Sigma–Aldrich) at 37 °C in a 5% CO_2_ incubator.

### Immunofluorescence

Oocytes were fixed in 4% paraformaldehyde (PFA) for 30 min, followed by permeabilization in 0.3% Triton X‐100 in phosphate‐buffered saline for another 30 min, and subsequently blocked with a blocking buffer for 30 min before primary antibody incubation. Antibody staining was conducted according to standard protocols as previously described.^[^ ^29^ ^]^ Details of the antibodies used are provided in Table S6 (Supporting Information). Images were acquired using an LSM710 confocal microscope (Zeiss, Jena, Germany), and fluorescence signal intensity was analyzed semi‐quantitatively with ImageJ software (NIH, Bethesda, MD, USA).

### Western Blotting

Oocytes were lysed in β‐mercaptoethanol buffer, heated at 95 °C for 10 min and analyzed by Sodium dodecyl sulfate‐polyacrylamide gel electrophoresis (SDS‐PAGE) and western blot.^[^ ^39^ ^]^

### HPG Staining

Oocytes were incubated in M16 medium with 100 mm HPG for 1 h and then fixed in 4% formaldehyde for 30 min. HPG incorporation was detected using the Click‐iT Cell Reaction Kit (Life Technologies), and the average cytoplasmic signal was quantified using ImageJ software.

### PAIso‐seq Library Construction

Construction of PAIso‐seq libraries (WT GV, WT MI oocytes, cKO GV, and cKO MI oocytes, 100 oocytes per sample) followed a previously published protocol. Briefly, total RNA was extracted using TRIzol reagent (Invitrogen, #15596026) and chloroform (Sinopharm, #10006818), precipitated with isopropanol (Sinopharm, #80109218), and dissolved in nuclease‐free water. Purified RNA was used for end extension with dU‐containing oligos and Klenow fragment 3′‐5′ exo^−^ (NEB, #M0212L). Template RNA was purified using the USER enzyme (NEB, #M5505S) and RNA Clean & Concentrator‐5 Kit (Zymo Research, #R1016). Purified template RNA was reverse‐transcribed into cDNA using SuperScript II reverse transcriptase (Invitrogen, #18064‐014) and TSO primers. Full‐length cDNA was further pre‐amplified using 2×KAPA HiFi HotStart ReadyMix (KAPA Biosystems, #KK2601) and cleaned using SPRIselect beads (Beckman Coulter, #B23318). For each sample, 20 ng of purified cDNA was used for the final amplification and cleaning as a pre‐amplification step. Finally, cDNAs from different samples were mixed according to their concentration and fragment length, and 500 ng of the mixed cDNA was used for the SMRTbell template library and sequenced using PacBio Sequell II.^[^ ^36^, ^37^ ^]^

### PAIso‐seq Data Processing

Sequenced reads were processed as previously described.^[^ ^5^ ^]^ Briefly, the subreads were converted into circular consensus sequencing (CCS) reads using the CCS software (v 6.4.0), and the CCS reads were further converted into FASTQ files using Bedtools (v 2.30.0). The FASTQ files were then split into samples by barcodes and trimmed into clean reads, which were aligned to the mm10 genome using minimap2 (v 2.24). Poly (A) information was extracted using scripts applied in a previous study.

### RNA‐seq Library Preparation

Oocytes from the specified genotypes (10 oocytes per sample) were collected, lysed immediately in 4.2 µL lysis buffer (containing 0.2% Triton X‐100, RNase inhibitor, deoxyribonucleotide triphosphate (dNTPs), oligo‐dT primers, and 1:1000 ERCC spike‐in), and used for cDNA synthesis via the Smart‐seq2 method.^[^ ^40^ ^]^ Sequencing libraries were prepared from 500 pg of amplified cDNA using the TruePrep DNA Library Prep Kit V2 for Illumina (catalog number: TD503, Vazyme, Nanjing, China) in accordance with the manufacturer's protocol. Barcoded libraries were pooled and sequenced on the HiSeq X Ten platform (Illumina, San Diego, CA, USA) with 150 bp paired‐end reads.

### RNA‐seq Analysis

RNA‐seq was performed using three independent biological replicates per sample. Raw sequencing reads were processed by trimming to 50 bp and aligned to the mouse genome (mm10) and ERCC spike‐in sequences using the TopHat v2.1.1. Transcripts were assembled from uniquely mapped reads based on reference annotations from University of California Santa Cruz (UCSC) gene models with Cufflinks v2.2.1. Transcript expression was quantified in FPKM, normalized to the ERCC spike‐in controls. Only transcripts with an FPKM greater than 1 in at least one sample were considered for analyses. Functional annotation was conducted via DAVID (https://david.ncifcrf.gov/tools.jsp). Spearman correlation coefficient (rs) was calculated using the “cor” function. Additional RNA‐seq data utilized in this study are summarized in Tables S1–S5 (Supporting Information).

### Statistical analysis

Data are expressed as the mean ± standard error of the mean. Group comparisons were performed using a two‐tailed unpaired Student's *t*‐test. Statistical significance was defined as *p* < 0.05, *p* < 0.01, and *p* < 0.001, denoted by ^*^, ^**^, and ^***^, respectively. “n.s.” indicates non‐significant. Most of the experiments used at least three independent samples and were repeated at least three times. Statistical analysis was carried out using GraphPad Prism.

## Conflict of Interest

The authors declare no conflict of interest

## Author Contributions

Y.F. conceived the study. H.Y.F., X.X.D., Y.K.W., and S.B.P. designed and analyzed the experiments. X.X.D., Y.K.W., S.B.P., F.J.H., Y.W.W., H.Q., Z.Y.J., and L.W.Z. performed the experiments. H.Y.F. and X.X.D. prepared the manuscript. X.X.D., Y.K.W., S.B.P., Z.L. performed the data analysis, and F.J.H. contributed equally to this study. Y.W.W., S.B.P., and F.J.H. are co‐first authors.

[Correction added on 07 May 2025, after first online publication: author Zhiyi Li (Z.L.) has been added in this version.]

## Supporting information



Supporting Information

Supplementary Table 2

Supplementary Table 5

## Data Availability

RNA‐seq and PAIso‐seq data have been deposited in the NCBI Gene Expression Omnibus database under the accession code GSE184028 and GSE276390, respectively.
