# Supplementary material for: PABPN1 Couples the Polyadenylation and Translation of Maternal Transcripts to Mouse Oocyte Meiotic Maturation
Source: Adv Sci (Weinh). 2025 Apr 23;12(23):2500048. doi: 10.1002/advs.202500048 (PMC12199313; doi:10.1002/advs.202500048)
Supplement: Supplementary file 1 — Supporting Information [file ADVS-12-2500048-s003.docx]

**Supplementary Materials**

**Supplementary figures**


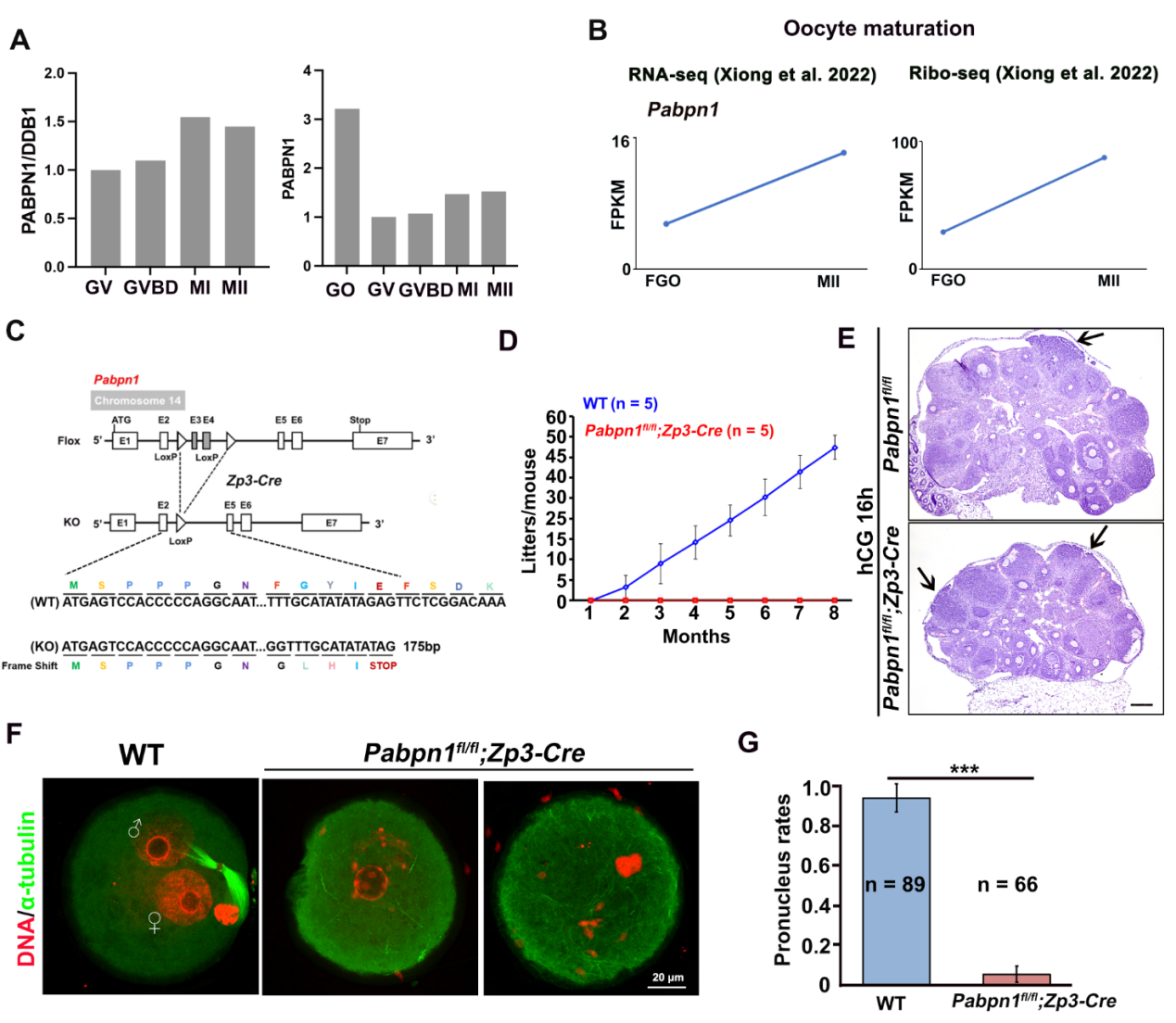


**Fig. S1. Phenotype analysis of WT and *Pabpn1^fl/fl^;Zp3-Cre* mice.**

**A:** Relative expression levels of PABPN1 and DDB1 across different meiotic stages in oocytes (left). Quantitative fluorescence analysis of PABPN1 (right). **B:** Transcriptional and translational dynamics of PABPN1 during oocyte maturation. **C:** A schematic representation of the strategy employed to generate *Pabpn1* conditional knockout mice. **D:** Fertility testing was performed on WT and *Pabpn1^fl/fl^;Zp3-Cre* mice, with 5 female mice included in each genotype group. **E:** H&E staining of ovarian sections from WT and *Pabpn1^fl/fl^;Zp3-Cre* mice. Scale bar = 100 μm. **F:** Confocal microscopy images depicting representative zygotes from WT and *Pabpn1^fl/fl^;Zp3-Cre* mice. Scale bar = 20 μm. **G:** Quantification of the pronucleus formation rate in zygotes (**F**). Data are expressed as mean ± SEM. Statistical significance was assessed using two-tailed Student’s *t* tests (****P* < 0.001). The number of zygotes analyzed is indicated (n).


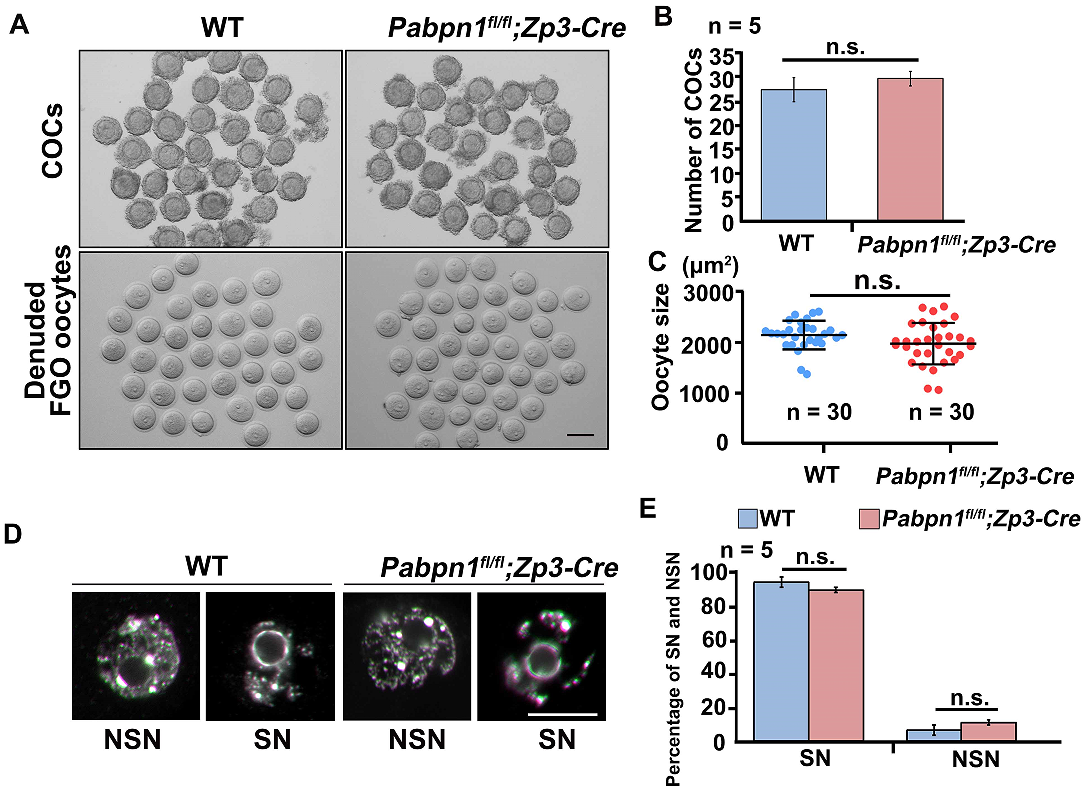


**Fig. S2.** **COCs analysis of WT and *Pabpn1^fl/fl^;Zp3-Cre* female mice.**

**A-B:** Representative images (**A**) and quantification (**B**) of COCs and denuded oocytes from antral follicles of WT and *Pabpn1^fl/fl^;Zp3-Cre* mice. Scale bar = 100 µm. Data are expressed as mean ± SEM. n.s.: non-significant. Sample size (n) is indicated. **C:** Quantitative analysis of the WT and *Pabpn1*-null oocytes. n: Number of analyzed oocytes. Error bars represent SEM. n.s. indicates no significant difference. **D:** Immunofluorescence analysis depicting oocytes with either a non-surrounded nucleolus (NSN) or a surrounded nucleolus (SN) as shown in (**A**). Scale bar, 20 µm. **E:** The proportion of oocytes exhibiting either a SN or a NSN in (**D**). Error bars represent SEM. n.s. indicates no significant difference. The sample size (n) refers to the number of mice analyzed.


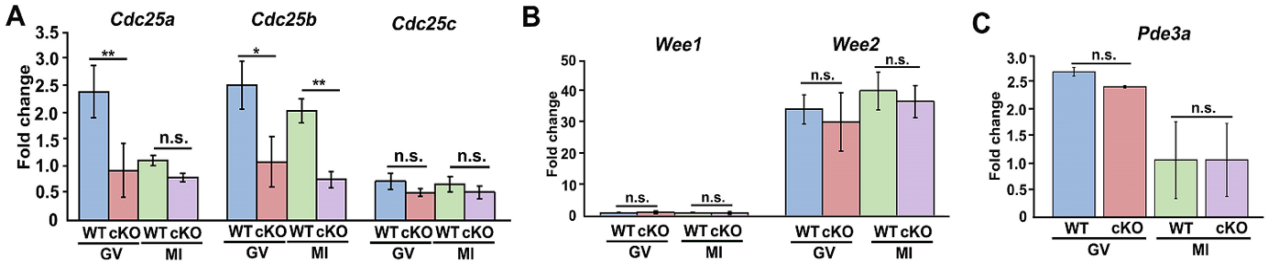


**Fig. S3. The expression levels of the specified transcripts in WT and *Pabpn1*-deficient oocytes.**

**A:** Relative levels of *Cdc25a,* *Cdc25b,* and *Cdc25c* in WT and *Pabpn1*-null oocytes were measured using RT-qPCR. Error bars represent SEM. Statistical analysis was assessed using Student’s *t*-test. **P* < 0.05, ***P* < 0.01. n.s.: non-significant. **B-C:** Quantitative RT-PCR results showing the relative levels of *Wee1, Wee2,* and *Pde3a* in WT and *Pabpn1-*null oocytes at the GV and MII stages. Error bars, SEM. Statistical analysis was performed using the Student’s *t*-test. n.s.: non-significant.


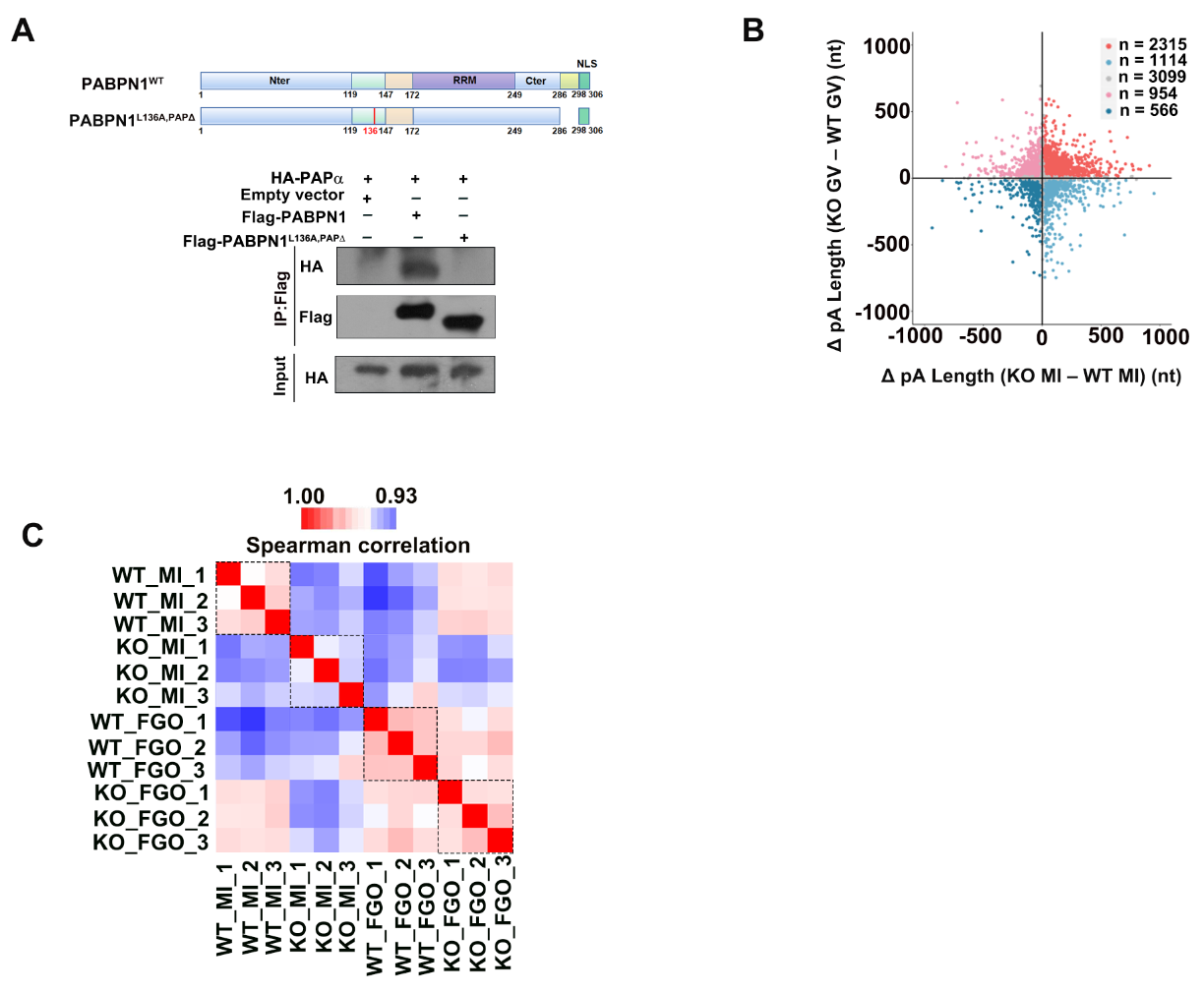


**Fig. S4. Analysis of polyadenylation in oocytes of *Pabpn1^fl/fl^;Zp3-Cre* and WT mice.**

**A:** Immunoprecipitation results revealed an interaction between PABPN1 and the polyadenylation enzyme PAPα. **B:** Scatter plot comparing poly(A) length changes between WT, *Pabpn1*-null GV oocytes (KO/WT), and MI oocytes (KO/WT). **C:** Heatmap displaying the Spearman’s correlation coefficients for all transcripts between WT and *Pabpn1*-null oocytes at both GV and MI stages.

**Supplementary Tables**

**Table S1. Spearman correlation coefficients among WT and *Pabpn1*-null growing oocytes*.***

|  |  | **WT FGO** | | | ***Pabpn1^fl/fl^; Zp3-Cre* mice FGO** | | | **WT MI** | | | ***Pabpn1^fl/fl^; Zp3-Cre* mice MI** | | |
| --- | --- | --- | --- | --- | --- | --- | --- | --- | --- | --- | --- | --- | --- |
|  | **Sample** | **1** | **2** | **3** | **1** | **2** | **3** | **1** | **2** | **3** | **1** | **2** | **3** |
| **WT FGO** | **1** | 1 | 0.960 | 0.965 | 0.938 | 0.940 | 0.954 | 0.965 | 0.963 | 0.965 | 0.932 | 0.945 | 0.950 |
|  | **2** | 0.960 | 1 | 0.967 | 0.946 | 0.942 | 0.947 | 0.964 | 0.964 | 0.964 | 0.928 | 0.935 | 0.945 |
|  | **3** | 0.965 | 0.967 | 1 | 0.945 | 0.944 | 0.952 | 0.967 | 0.967 | 0.965 | 0.940 | 0.942 | 0.952 |
| ***Pabpn1^fl/fl^; Zp3-Cre* FGO** | **1** | 0.938 | 0.946 | 0.945 | 1 | 0.957 | 0.953 | 0.943 | 0.942 | 0.954 | 0.941 | 0.945 | 0.954 |
|  | **2** | 0.940 | 0.942 | 0.944 | 0.957 | 1 | 0.952 | 0.940 | 0.940 | 0.945 | 0.938 | 0.945 | 0.956 |
|  | **3** | 0.954 | 0.947 | 0.952 | 0.953 | 0.952 | 1 | 0.954 | 0.953 | 0.956 | 0.943 | 0.956 | 0.966 |
| **WT MI** | **1** | 0.965 | 0.964 | 0.967 | 0.943 | 0.940 | 0.954 | 1 | 0.965 | 0.964 | 0.965 | 0.966 | 0.966 |
|  | **2** | 0.963 | 0.964 | 0.967 | 0.942 | 0.940 | 0.953 | 0.965 | 1 | 0.970 | 0.958 | 0.966 | 0.959 |
|  | **3** | 0.965 | 0.964 | 0.965 | 0.954 | 0.945 | 0.956 | 0.964 | 0.970 | 1 | 0.965 | 0.970 | 0.965 |
| ***Pabpn1^fl/fl^; Zp3-Cre* MI** | **1** | 0.932 | 0.928 | 0.940 | 0.941 | 0.938 | 0.943 | 0.965 | 0.958 | 0.965 | 1 | 0.971 | 0.969 |
|  | **2** | 0.945 | 0.935 | 0.942 | 0.945 | 0.945 | 0.956 | 0.966 | 0.966 | 0.970 | 0.971 | 1 | 0.969 |
|  | **3** | 0.950 | 0.945 | 0.952 | 0.954 | 0.956 | 0.966 | 0.966 | 0.959 | 0.965 | 0.969 | 0.969 | 1 |

**Table S2. Poly(A) tail length of transcripts in GV and MI oocytes from WT and *Pabpn1^fl/fl^; Zp3-Cre* mice. (In a separate xlsx file).**

**Table S3. Primer sequences.**

| **Primer**  **name** | **Genes**  **targeted** | **Application** | **Sequences (5**′**-3**′**)** |
| --- | --- | --- | --- |
| *Copa*-CDS-qt-F | *Copa* | Real-time PCR | 5′- ACCAAATTCGAGACGAAGAGC -3′ |
| *Copa*-CDS-qt-R | *Copa* | Real-time PCR | 5′- CCGATAGTCCCATAACTGGATGA -3′ |
| *Ccnb3-*CDS-qt-F | *Ccnb3* | Real-time PCR | 5′- AGAGAAACCTGTGATTCAGGAGA -3′ |
| *Ccnb3-*CDS-qt-R | *Ccnb3* | Real-time PCR | 5′- GAAATGCGGCTTTTTGACTGG -3′ |
| *Zfp60-*CDS-qt-F | *Zfp60* | Real-time PCR | 5′- ATCATGGTGAAAAAGCCTTGTCT -3′ |
| *Zfp60-*CDS-qt-R | *Zfp60* | Real-time PCR | 5′- AAGTGCTGTAACTCAAGAGAACC -3′ |
| *Kit-*CDS-qt-F | *Kit* | Real-time PCR | 5′- CTCCCCCAACAGTGTATTCAC -3′ |
| *Kit-*CDS-qt-R | *Kit* | Real-time PCR | 5′- TAGCCCGAAATCGCAAATCTT -3′ |
| *Mos-*CDS-qt-F | *Mos* | Real-time PCR | 5′- TGGCTGGTTTTGAGAATCAAGG -3′ |
| *Mos-*CDS-qt-R | *Mos* | Real-time PCR | 5′- GTCACATGAGACACTAGGGAGA -3′ |
| *Wee1-*CDS-qt-F | *Wee1* | Real-time PCR | 5′- GTCGCCCGTCAAATCACCTT -3′ |
| *Wee1-*CDS-qt-R | *Wee1* | Real-time PCR | 5′- GAGCCGGAATCAATAACTCGC -3′ |
| *Cdc25b-*CDS-qt-F | *Cdc25b* | Real-time PCR | 5′- TCCGATCCTTACCAGTGAGG -3′ |
| *Cdc25b-*CDS-qt-R | *Cdc25b* | Real-time PCR | 5′- GGTCTCTGGAAGCGCACATT -3′ |
| *Btg4-pat* | *Btg4* | Pat assay | 5′- GTAGGTTTTCAACTAAGGAAGAT -3′ |
| *Lypla1-pat* | *Lypla1* | Pat assay | 5′- CTAGATAGAATGAAGCCAAATG -3′ |
| *Tcea1-pat* | *Tcea1* | Pat assay | 5′- TCTACAGAGTGAGTTCCAGGAC -3′ |
| *Ccnb1-pat* | *Ccnb1* | Pat assay | 5′- GTGTGCTTTGAATTCTGACAGCCA -3′ |
| *Cdk7-pat* | *Cdk7* | Pat assay | 5′- GAAGAGGCCTCGGGGAGATTTG -3′ |

**Table S4. Quality control of RNA-seq results (growing oocytes from WT and *Pabpn1^fl/fl^; Zp3-Cre* mice).**

| **Sample** | **Total reads** | **Mapping efficiency** |
| --- | --- | --- |
| WT-FGO-Rep1 | 23,329,460 | 88.5% |
| WT-FGO-Rep2 | 25,244,986 | 90.1% |
| WT-FGO-Rep3 | 20,620,367 | 80.9% |
| *Pabpn1*-FGO-Rep1 | 29,524,507 | 85.7% |
| *Pabpn1*-FGO-Rep2 | 35,532,876 | 91.2% |
| *Pabpn1*-FGO-Rep3 | 32,486,662 | 92.3% |
| WT-MI-Rep1 | 18,090,196 | 91.70% |
| WT-MI-Rep2 | 20,693,176 | 89.20% |
| WT-MI-Rep3 | 21,967,355 | 89.00% |
| *Pabpn1*-MI-Rep1 | 12,590,566 | 85.6% |
| *Pabpn1*-MI-Rep2 | 19,904,212 | 87.4% |
| *Pabpn1*-MI-Rep3 | 19,837,646 | 88.5% |

**Table S5. FPKMs of RNA-seq results (in a separate xlsx file).**

**Table S6. Antibody information.**

| **Protein**  **name** | **Manufacture**  **(catalogue number)** | **Applications**  **(working dilution)** | **Website Link** |
| --- | --- | --- | --- |
| **PABPN1** | Abcam (ab86209) | WB (1:500) | <https://www.abcam.com/cnot6-antibody-ab86209.html> |
| **DDB1** | Epitomics (3821-1) | WB (1:1000) | http://www.epitomics.com/products/search/DDB1 |
| **Phalloidine** | Invitrogen (R415) | IF (1:400) | https://www.thermofisher.cn/order/catalog/product/R415?SID=srch-srp-R415 |
| **α-Tubulin** | Sigma (F2168) | IF (1:200) | https://www.sigmaaldrich.cn/CN/zh/product/sigma/f2168 |
| **CREST** | Fitzgerald Industries International (70R-21494) | IF (1:100) | https://www.fitzgerald-fii.com/crest-antibody-70r-21494.html |
| **Pericentrin** | BD transduction laboratories  (611814) | IF (1:300) | http://www.bdbiosciences.com/cn/reagents/research/antibodies-buffers/cell-biology-reagents/cell-biology-antibodies/purified-mouse-anti-mouse-pericentrin-30pericentrin/p/611814 |
| **TPX2** | Novus Biologicals (NB500-183) | IF(1:200)  WB (1:2000) | https://www.novusbio.com/products/tpx2-antibody_nb500-183 |
| **BUB3** | Santa Cruz (sc-28258) | IF (1:200) | https://www.scbt.com/scbt/zh/product/bub3-antibody-h-10 |
| **TOP2B** | Abcam (ab109524) | IF (1:200) | http://www.abcam.com/Topoisomerase-II-beta-antibody-EPR5377-ab109524.html |
| **HA** | Cell Signaling (3724) | IF (1:400) | https://www.cellsignal.com/products/primary-antibodies/ha-tag-c29f4-rabbit-mab/3724 |
| **p^T14/Y15^CDK1** | Invitrogen (701808) | IF (1:400)  WB (1:1000) | https://www.thermofisher.cn/cn/zh/antibody/product/Phospho-CDK1-Thr14-Tyr15-Antibody-clone-17H29L7-Recombinant-Monoclonal/701808 |
| **p^S22^Lamin A/C** | Cell Signaling (2026) | IF (1:400)  WB (1:1000) | https://www.cellsignal.cn/products/primary-antibodies/phospho-lamin-a-c-ser22-antibody/2026?site-search-type=Products&N=4294956287&Ntt=lamin+a%2Fc&fromPage=plp |
| **Cyclin B1** | Cell Signaling (4138) | WB (1:1000) | https://www.cellsignal.cn/products/primary-antibodies/cyclin-b1-antibody/4138?site-search-type=Products&N=4294956287&Ntt=4138&fromPage=plp&_requestid=3272799 |
| **CPEB1** | Proteintech (13274-1-AP) | WB (1:1000) | https://www.ptglab.com/products/CPEB1-Antibody-13274-1-AP.htm |
| **CDC25B** | Abcam  (ab124819) | WB (1:1000) | https://www.abcam.cn/products?keywords=cdc25b |
| **CDK1** | Abcam  (ab133327) | WB (1:1000) | https://www.abcam.cn/cdk1-antibody-epr165-ab133327.html |
| **PAPα** | Sigma-Aldrich (HPA001788) | IF (1:100) WB (1:1000) | https://www.sigmaaldrich.cn/CN/zh/search/(hpa001788)?focus=products&page=1&perpage=30&sort=relevance&term=%28hpa001788%29&type=product |
| **Flag** | Sigma (F3165) | WB (1:3000) | http://www.sigmaaldrich.com/catalog/product/sigma/f3165?lang=zh&region=CN |
